# Human Milk Metabolic Hormones: Analytical Methods and Current Understanding

**DOI:** 10.3390/ijms22168708

**Published:** 2021-08-13

**Authors:** Majed A. Suwaydi, Zoya Gridneva, Sharon L. Perrella, Mary E. Wlodek, Ching Tat Lai, Donna T. Geddes

**Affiliations:** 1 School of Molecular Sciences, The University of Western Australia, Crawley, WA 6009, Australia; majed.suwaydi@research.uwa.edu.au (M.A.S.); sharon.perrella@uwa.edu.au (S.L.P.); mary.wlodek@uwa.edu.au (M.E.W.); ching-tat.lai@uwa.edu.au (C.T.L.); donna.geddes@uwa.edu.au (D.T.G.); 2 Department of Medical Laboratory Technology, College of Applied Medical Sciences, Jazan University, Jazan 54142, Saudi Arabia; 3 Population Health, Murdoch Children’s Research Institute (MCRI), Parkville, VIC 3052, Australia

**Keywords:** leptin, adiponectin, ghrelin, insulin, obestatin, resistin, apelin, human milk, analytical methods, metabolic hormones

## Abstract

Human milk (HM) contains a wide array of peptide hormones including leptin and adiponectin, which are involved in the regulation of infant growth and development. These essential hormones might play an important role in the regulation of metabolic reprogramming of the new-born infant. However, HM hormone studies are sparse and heterogeneous in regard to the study design, sample collection, preparation and analysis methods. This review discussed the limitations of HM hormone analysis highlighting the gaps in pre-analytical and analytical stages. The methods used to quantify HM metabolic hormones (leptin, adiponectin, ghrelin, insulin, obestatin, resistin and apelin) can be classified as immunoassay, immunosensor and chromatography. Immunoassay methods (ELISA and RIA) have been predominantly used in the measurement of these HM hormones. The relative validity parameters of HM hormones analysis are often overlooked in publications, despite the complexity and differences of HM matrix when compared to that of plasma and urine. Therefore, appropriate reports of validation parameters of methodology and instrumentation are crucial for accurate measurements and therefore better understanding of the HM metabolic hormones and their influences on infant outcomes.

## 1. Introduction

Human milk (HM) is a unique heterogeneous specific mixture of essential macronutrients and micronutrients along with hormones, immunoglobulins and other bioactive molecules [1] that provide the optimal source of nutrition for infants born at term and, when fortified, for preterm infants. Thus, breastfeeding confers both short- and long-term benefits to infants including a reduced risk of chronic diseases [2]. The protective activity of HM is thought to be due to the presence of bioactive molecules that are dynamic in response to maternal conditions and can positively modulate energy metabolism and inflammatory responses in infants and mothers [3].

Hormones such as leptin, adiponectin, ghrelin, insulin, resistin, obestatin and apelin have been recently identified in HM. Existing evidence supports the role of these HM hormones in programming the new-born infant’s metabolism [4,5,6]. Indeed, these HM hormones are implicated in the regulation of infant growth and development of body composition [3,4,7] and likely contribute to the reduced risk of obesity and diabetes in children and adults that have been breastfed in infancy [8,9]. A detailed summary of associations of these HM hormones with maternal adiposity and infant outcomes is provided in Supplementary Tables S1–S7.

With increasing interest in HM hormones and their relationships to infant growth and development, it is important to review the available evidence and discuss current measurement methods in HM. Hormone measurement protocols for HM are often adapted from assays designed for other body fluids such as urine and plasma. Assay optimisation is a mandatory step as milk contains significant amounts of fat and carbohydrates that may influence the chemical and physical behaviour of the samples. Furthermore, HM composition studies have shown complexity considering diurnal and circadian rhythms. Despite this, consideration of the variations related to inter-feed intervals, pre- and post-feed sampling and circadian rhythms and dose-effects is still lacking in HM hormones analysis. In addition, maternal milk production is another important concept of analysing HM hormone, thus dose and intake of HM hormones received by infant might be a key for biological impact. In this review, we present the current knowledge and methodological approaches for analysing hormones in HM.

## 2. Results and Discussion

### 2.1. Leptin

Leptin, a polypeptide hormone with an average of 167 amino acids (16.2 kDa), is mainly synthesised and secreted by adipocytes of white adipose tissue as a product of the obese (Ob) gene expression [10]. Leptin signals the amount of fat stored in the body; thus, plasma leptin concentration is positively correlated with body fat mass in adults [11,12]. Leptin modulates energy homeostasis; it induces energy consumption and reduces food intake by initiating a signal cascade beginning with leptin binding to the Ob receptor in the hypothalamus [13,14]. At birth, cord blood leptin concentrations are reported to correlate positively with neonatal fat mass index [15]. Two mechanisms contribute to the presence of leptin in HM. First, it is suggested that maternal plasma leptin enters HM via diffusion or receptor-mediated transport [16,17,18]. Second, mammary epithelial cells can produce small amounts of leptin [19]. HM leptin is present in a relatively high and variable concentration (range: 0.2 to 1.47 ng/mL), which generally decreases as the duration of lactation increases (Table 1).

#### 2.1.1. Analytical Methods

Typically, enzyme-linked immunosorbent assays (ELISA) and radioimmunoassay (RIA) are the methods that predominantly have been used to measure HM leptin (Table 1). Out of 50 studies, 30 (60%) used ELISA and 15 (30%) used RIA. However, new measurement methods have recently been employed. Ojeda et al. [40] developed an electrochemical immunosensor method (ECIs) utilising a magneto immunosensor. This method involves immobilisation of a specific biotinylated anti-leptin antibody on the surface of streptavidin-functionalised magnetic beads and a sandwich-type immunoassay involving the target analyte, monoclonal anti-leptin and IgG labelled with alkaline phosphatase (AP-IgG) that is trapped onto screen-printed carbon electrodes (electrochemical transduction). The ECIs showed better analytical sensitivity than that reported for other immunoassays. Calibration plot linearity was achieved between 0.5 and 100 pg/mL with a detection limit (LOD) of 0.5 pg/mL. The ECIs method also required a standard addition method to avoid the matrix effects of HM; thus, it is more expensive than other methods, possibly explaining why it has not been widely adopted [40].

A magnetic bead-based multiplex immunoassay has also been designed to measure multiple analytes in HM [46]. This method is attractive with simultaneous measurement of multiple biomarkers in a single run using as little as 12.5 to 50 μL of milk. The principle of multiplex immunoassay is analogous to that of the sandwich ELISA where capture antibodies (specific for desired analytes) coupled to beads react with a sample that contains the analytes. The resulting complexes react with a biotinylated detection antibody-forming sandwich complex that is detected by fluorescent methods [68]. For HM analysis, the assay performance parameters (LOD, and % coefficient of variation (CV)) reported by Andreas et al. [46] were promising. However, the measured HM leptin concentration was ~100-fold (575 ng/mL) higher than the reported concentrations using RIA [44], ELISA [18], and multiplex assay [69]. Currently, this method cannot be directly compared to ELISA as the recovery of the assay has not been reported (Table 1).

Another recently developed analytical method is based on immunoaffinity purification followed by analysis with nano-high pressure liquid chromatography coupled with a high-resolution mass spectrometry analyser (nano-HPLC-HRMS) [65]. The nano-HPLC-HRMS reported a LOD of 27 ng/mL, which was substantially higher than the LOD of RIA and ELISA (0.017 and 0.05 ng/mL, respectively) [18,44]. Further, the average concentration of HM leptin reported by nano-HPLC-HRMS (6.7 ng/mL) is much higher than values reported by RIA and ELISA and below the detection limit reported by the authors (LOD = 27 ng/mL) [65].

#### 2.1.2. Sample Preparation

RIA has been primarily used to measure HM leptin in skim milk because triglycerides in whole milk interfere with the binding of radioactive-labelled antigens to antibodies, thus compromising assay sensitivity [23]. As an alternative, ELISA has been successfully optimised to measure leptin in whole milk [18,29,47,48], yet most studies still measure leptin in skim milk (Table 1). This may lead to erroneous observed relationships as HM leptin is reported to be up to 50% higher in whole HM compared to skim HM [48]. It has been previously assumed that higher whole milk leptin concentrations are due to the lipophilic nature of leptin, however, leptin concentrations reportedly have no association with HM fat content and do not differ between pre- and post-feed samples despite increasing HM fat content over the course of a breastfeed [48,70]. Nevertheless, precipitation of HM lipids, before the immunoaffinity extraction was not achieved without precipitating leptin [65], which warrants further investigation.

#### 2.1.3. Maternal Influences on HM Leptin

In general, a positive association between maternal adiposity and HM leptin concentration has been established for skim and whole milk [7,18,36,38,46], however Khodabakhshi et al. [43] have reported that relationship only in mothers with obesity (as provided by author on request).

There are limited data regarding associations between leptin and maternal health conditions such as gestational diabetes mellitus (GDM). Nunes et al. [54] reported that HM leptin concentrations in women with GDM decreased from colostrum to mature milk [54]. Similarly, Yu et al. [57] reported women with GDM had significant reduction in HM leptin concentrations over time; median concentration of 1.28, 0.26 and 0.20 pg/mL were measured at 3, 42 and 90 days of lactation, respectively. However, these outcomes were only investigated in skim milk, which reported a significantly lower concentration of leptin in comparison with whole milk. Whilst maternal blood leptin concentrations follow a circadian pattern, only one HM study has shown a possible 24-h (24 h) pattern with skim milk leptin concentrations being significantly higher at night (22:00–04:00 h) [44]. These findings are yet to be confirmed in whole HM leptin. A summary of studies that have investigated HM leptin is presented in Table 1.

#### 2.1.4. HM Leptin and Infant Outcomes

The exact roles of HM leptin in infant development, particularly in early growth and body composition, are still unclear [71]. More recently, the 24 h intake of whole HM leptin was associated positively with infant adiposity over the first 12 months of lactation [7]. In contrast, skim HM leptin concentrations have shown negative [42,51] and no association [38,43] with infant body composition. Reported relationships between infant outcomes and skim milk leptin concentrations, rather than the infant’s HM leptin dose through whole milk intake should be interpreted with caution.

### 2.2. Adiponectin

Adiponectin, the most abundant adipokine, consists of 247-amino acids (27 kDa). Three circulating oligomers of adiponectin have been identified in the blood (low-, medium- and high- molecular weight), each with discrete biological activities. The high molecular weight oligomer is the more active and prevalent form of adiponectin in intracellular spaces, whereas low-molecular-weight adiponectin dominates the blood circulation [72]. Adiponectin is involved in the regulation of lipid and glucose metabolism and the stimulation of food intake, as well as the reduction of energy expenditure and regulation of the inflammatory response [3,73]. Although adiponectin is mainly synthesised by adipocytes, some evidence suggests that it also may be synthesised by the mammary epithelial cells [71]. Adiponectin is also the most abundant adipokine in HM, with a concentration range of ~4.2–78.9 ng/mL [7,74,75]. HM is dominated by the more biologically active high-molecular-weight adiponectin [76] that may enhance insulin sensitivity and metabolic control and suppresses inflammation [74].

#### 2.2.1. Analytical Methods

HM adiponectin is most commonly measured with immunoassays such as RIA and ELISA. Out of 36 studies, 26 (72%) used ELISA and 8 (22%) used RIA. Ozarda et al. [77] suggested using skim milk to measure HM adiponectin when using RIA, as the linearity and recovery of skim milk assay are better in comparison with whole milk, although, the assay’s linearity and recovery values were not reported in the manuscript. Ley et al. [78], measured HM adiponectin using RIA in both skim and whole milk and reported an improved assay recovery when using whole milk (whole milk: 105 ± 18%, skim milk: 118 ± 36%). Measurement of whole milk adiponectin using the ELISA assay has also been established and validated [28]. The direct comparison of HM adiponectin RIA and ELISA assays performance considering assay recovery showed that ELISA assay is most likely the best practice for measurement of HM adiponectin. That is, out of 8 studies that measured HM adiponectin using RIA, only 3 studies reported less acceptable recovery values in comparison with ELISA assay recovery values (Table 2). Two studies used DELFIA (DELFIA immunoassay), an alternative to ELISA that has a stable time-resolved fluorescent signal which improves the assay dynamic range and a multiplexing capability, however reported similar to ELISA LOD (0.8 ng/mL) [79,80].

#### 2.2.2. Sample Preparation

Although measuring HM adiponectin in skim milk is a common practice (Table 2), the measurement method for whole HM adiponectin has been established and validated [18,28], as it is more representative of the milk consumed by the infant. Only one study compared skim and whole HM adiponectin concentrations and, unlike leptin, found no significant difference [82]. Additionally, it is important to note that the mean of assay recovery values of skim milk (118.4 ± 36%) was less acceptable in comparison with the recovery value of whole milk (105.5 ± 18.2%). Pre- and post-feed samples showed no significant differences in adiponectin concentration when measured in whole milk [18], indicating acceptability of skim milk adiponectin results.

#### 2.2.3. Maternal Influences on HM Adiponectin

HM adiponectin concentrations have been shown to be positively associated with maternal serum adiponectin concentrations [17,85,86,88], which in turn are negatively related to maternal weight and BMI [91,92]. Andreas et al. [93] reviewed nine studies to assess the association of maternal BMI with adiponectin levels in HM. They concluded that only two studies [75,76] showed a positive relationship with maternal BMI that disappeared after adjusting for time postpartum.

#### 2.2.4. HM Adiponectin and Infant Outcomes

Higher adiponectin levels in HM appear to be associated with reduced infant weights and lengths up to 6 months of age [76]. Although some studies report a positive correlation between HM adiponectin and infant anthropometrics after 4–6 months [41,42,86,87], others have failed to find a relationship [56,81,83]. The interpretation of these findings is complicated by the fact that there is no standard protocol for the collection and analysis of HM samples. Moreover, the mechanisms by which HM adiponectin impacts the infant maybe more complex than currently believed. Most studies have examined the HM concentration of adiponectin rather than actual infant intake. Gridneva et al. [7] have shown that higher HM adiponectin intake was associated with lower infant lean mass and high adiposity during the first 12 months of lactation.

### 2.3. Ghrelin

Ghrelin is a small polypeptide hormone comprising 28-amino acids (33.7 kDa) and is predominantly synthesised by the stomach, with smaller amounts produced by other organs, including the pancreas, kidneys and placenta [94]. Two different isoforms of ghrelin exist in the circulation: acetylated ghrelin (active) and deacylated ghrelin (restricted activity). Ghrelin is an appetite-stimulant hormone; it acts in a positive feedback loop that signals feeding behaviour to the hypothalamus, inducing food intake and controlling energy homeostasis. Further, in humans active ghrelin induces growth hormone (GH) secretion through binding and activation of GH secretagogue receptor 1a (GHS-R 1a) [95]. Ghrelin is present in HM, and there are two potential sources, maternal circulating ghrelin [31,81,96] and mammary epithelial cell expression [97,98].

#### 2.3.1. Analytical Methods

Like adiponectin, different forms of HM ghrelin are mainly measured using enzyme immunoassays, RIA and ELISA. Out of 19 studies, 11 (58%) used RIA and 5 (26%) used ELISA. Most studies report HM ghrelin concentrations in skim milk with only two reports of ghrelin in whole milk (Table 3). Recently, high-pressure liquid chromatography (HPLC) and multiplex immune assays have been employed to measure HM ghrelin [46,99,100]. Aydin et al. [100] optimised a reverse-phase HPLC to perform a simultaneous quantification of acylated ghrelin and deacylated ghrelin. This method is considered fast, accurate and sensitive, and offers the advantage of simultaneous measurement of two forms of ghrelin. The LOD of this method was 11 ± 2 pg/mL for acylated ghrelin and 14 ± 3 pg/mL for deacylated ghrelin, and the recovery rate was similar to RIA (acylated ghrelin: 91%; deacylated ghrelin: 108%). The multiplex immunoassay of HM ghrelin has been used by Andreas et al. [46]; the following assay performance parameters were reported: lower limit of quantification (LLOQ) of 16.6 pg/mL, 1.2 pg/mL LOD and 4% CV. HM ghrelin concentrations reported by this method (Table 3) were considerably higher than that of RIA, ELISA and EIA with no validation of the protocol reported such as recovery or linearity of the assay. Moreover, Andreas et al. [46] reported HM ghrelin concentration (~60 ng/mL) that was considerably higher than that reported by Escuder-Vieco et al. (10.55–57.84 pg/mL) [69] who used the same method and reported results that were more consistent with RIA.

#### 2.3.2. Sample Preparation

A direct comparison between HM ghrelin concentration in skim and whole milk reported higher concentrations in whole milk and a positive correlation between HM ghrelin and fat content [98]. However, most studies have measured ghrelin in skim milk. Evaluation of the effects of sample collection protocols and storage conditions on measured ghrelin concentrations is only reported for blood [108]. It was found that the use of aprotinin as a proteases inhibitor and 1N HCl as an acidification agent improved the measurement of HM acylated-ghrelin (aGh) by protecting aGh from proteolytic activity [108]. In HM, a limited number of studies that measured ghrelin also treated samples using protease inhibitors and acidification agents [31,101,104]. However, the effects of using a protease inhibitor and acidification agent on ghrelin concentration have not been investigated. Reported HM ghrelin concentrations vary due to differences in assay methods and ghrelin isoform targets; total ghrelin (tGh), acylated-ghrelin (aGh) or deacylated-ghrelin (dGh) (Table 3). Thus, establishment of a standard collection, storage and analytical protocol for measurement of HM ghrelin is warranted as well as studies to determine 24 h variation of HM ghrelin concentration.

#### 2.3.3. Maternal Influence on HM Ghrelin

Some studies have reported a negative relationship between HM ghrelin and maternal BMI [41,96,102], and HM ghrelin concentrations are higher in the HM of mothers of infants with normal weight compared to mothers of infants with obesity (137.5 pg/mL and 132.0 pg/mL, respectively) [43]. The relationships between maternal BMI, HM ghrelin and infant growth may have a role in reducing the risk of obesity later in life. Moreover, HM ghrelin concentrations are reported to differ between mothers with and without GDM, with lower concentrations of ghrelin measured in the colostrum and mature milk of GDM mothers [57]. These findings are in conflict with a previous report that suggested the effect of GDM on HM ghrelin might be limited to colostrum as there were no detectable differences in ghrelin concentrations in mature milk between mothers with and without GDM [99].

#### 2.3.4. HM Ghrelin and Infant Outcomes

Infants receive early exposure to ghrelin through the placenta and HM suggesting a potential role of ghrelin in early programming; however, the evidence is not consistent [71,109]. HM active ghrelin concentration has been reported to correlate positively with infant growth rate and weight gain [83]. Further, when compared to breastfed infants, those fed formula have a higher weight and growth rate; Savino et al. suggested this might be a result of exposure to the higher amount of total ghrelin in formula milk [105]. However, no association was reported between total ghrelin concentrations measurement and infant head circumference and weight-for-height z-score (WHZ) [57]. Moreover, positive correlations between the 24 h milk intake of HM ghrelin (skim milk) and infant weight gain [41] and HM ghrelin concentrations and infant birth weight have been reported previously [81].

### 2.4. Insulin

The β cells of the pancreas synthesise insulin, a glucose controlling hormone that consists of 51 amino acids (5.8 kDa) [110]. Insulin is present in HM, with the primary source being maternal blood. The detection of artificial insulin in HM of women with type 1 diabetes suggests an active transport mechanism of HM insulin from the maternal circulation [111]. However, some evidence suggests that mammary epithelial cells can also produce insulin [112]. Colostrum concentrations of insulin are substantial (114–306 mU/L) and rapidly decrease so HM concentrations match that of maternal blood by day 5 postpartum [111].

#### 2.4.1. Analytical Methods

HM insulin has been measured with a variety of methods, with 16 studies up to date using ELISA (8/16, 50%), RIA (3/16, 19%), electrochemiluminescence immunoassays (ECLIA; 2/16, 13%), chemiluminescent microparticle immunoassay (CMIA) and multiplex immune assay (Table 4). CMIA is a modified and advanced form of the ELISA technique that uses paramagnetic microparticles anti-insulin antibody-coated and acridinium-labelled insulin conjugate to form a microparticle–insulin–conjugate sandwich. Then, the complex is pre-triggered by the hydrogen peroxide reagent and triggered by sodium hydroxide reagent to result in a chemiluminescent signal that read and used to interpret the insulin concentration in biological samples [113]. The CMIA method is reported to have acceptable recovery (93.78 ± 6.18%) and assay performance parameters (% CV or LOD or both—general approximates) that are comparable to other immunoassays (RIA and ELISA) (Table 4). The ECLIA insulin detection system uses two monoclonal antibodies that are specific for human insulin. Samples are incubated with biotinylated insulin-specific antibody and insulin-specific antibody labelled with a ruthenium complex to form a sandwich complex. The chemiluminescence reaction for detection of the sandwich complex is initiated by applying a voltage to the sample solution resulting in a precisely controlled reaction. The median concentration of insulin in colostrum reported by this method is 162 ± 64.2 pmol/L (~23.4 mU/L), which is comparable to that reported by Whitmore et al. [111] and Grunewald et al. [90] using ELISA (Table 4). However, the performance parameters of ECLIA HM insulin have not been reported.

#### 2.4.2. Sample Preparation

HM insulin concentrations have only been reported in skim milk with results indicating no significant differences between pre- and post-feed, and no changes over 24 h [111].

#### 2.4.3. Maternal Influence on HM Insulin

The relationship between HM insulin and maternal BMI has been reviewed [93]; of four studies, two reported negative relationships. Further, the positive relationship between maternal BMI and HM insulin is shown at 3 months postpartum [46]. More recently, a positive association was reported between HM insulin and maternal BMI [56,107] across the first 4 months of lactation. Mothers with GDM had a higher HM concentration of HM insulin in both colostrum and mature milk compared to those without GDM [57]. Ley et al. [78] also reported the same result for mature milk, but not colostrum.

#### 2.4.4. HM Insulin and Infant Outcomes

It has been speculated that the high concentration of HM insulin in response to higher maternal BMI might play a role in lowering infants’ body weight, weight-for-length z-score, BMI-for-age z-score and total lean mass at one month of age [36] and weight-for-length in the first year of life [56].

### 2.5. Resistin

Resistin is a 12.5 kDa cysteine-rich polypeptide hormones consisting of 114 amino acids. First discovered in rodents as an adipose-secreted hormone, resistin plays a physiological role as a subclinical inflammatory mediator between obesity and diabetes. Serum resistin concentrations are higher in obese and diabetic mice, and mouse models with diet-induced obesity [117]. Resistin antagonises insulin action and induces glucose intolerance in vivo [117]. In humans, the primary source of resistin is macrophages, therefore it is strongly related to inflammatory conditions [118]. Resistin gene expression has been reported in human placental tissue with its prominence in the chorionic villi during the first trimester suggesting a role in modulating insulin sensitivity during pregnancy [119]. Concentrations of resistin in HM are lower than that of maternal and infant serum, and decrease throughout the lactation period [120].

#### 2.5.1. Analytical Methods

Three studies to date have reported HM concentrations of resistin using immunoassays with skim milk (Table 5); two studies used ELISA [37,120], while the third study used multiplex immunoassay [46].

#### 2.5.2. Sample Preparation

Andreas et al. reported no difference in HM resistin concentrations within a feed, but the concentration decreased over lactation [46]. Similarly, Ilcol et al. showed HM resistin decreased gradually from colostrum to mature milk (up to 180 days postpartum) [120]. No data were available to compare differences between whole and skim milk concentrations of resistin.

#### 2.5.3. Maternal Influence on HM Resistin

No relationships between HM resistin and maternal anthropometrics have been reported [37,46].

#### 2.5.4. HM Resistin and Infant Outcomes

Savino et al. studied 41 term infants aged less than six months and found no relationship between HM resistin concentration and infant anthropometrics [37]. Infant intake of HM resistin has yet to be measured in relation to infant outcome.

### 2.6. Obestatin

Obestatin is a 23 amino acid peptide, discovered in 2003 as a carboxyl-terminal fragment of pro-ghrelin (C-ghrelin) [121]. Obestatin is encoded by the ghrelin gene and is derived from the carboxy-terminal part of proghrelin; ghrelin is derived from the N-terminal part of the same precursor preproghrelin. Obestatin is considered an antagonist to ghrelin as it inhibits feeding and digestive motility by binding to the orphan G protein-coupled receptor GPR39 [121,122]. Gronberg et al. [97] reported the subcellular localisation of obestatin and that obestatin immunoreactive cells were present in the gastrointestinal tract, pancreatic islets and mammary glands. Obestatin appears to be involved in many physiological functions, cell proliferation, decreasing food intake, reducing body weight gain, increasing the secretion of pancreatic enzymes, and inhibiting glucose-induced insulin secretion [123]. HM obestatin concentrations are 538.90 ± 46.21 pg/mL and 528.5 ± 39.00 pg/mL in HM colostrum and mature milk, respectively [31]. HM obestatin concentrations are almost twice higher than in maternal serum; indicating it is an important component for the infant that may be synthesised by the mammary gland.

#### 2.6.1. Analytical Methods

Obestatin concentrations have been measured in skim HM in two studies using RIA, and the results were comparable [31,92] (Table 6). The assay performance parameters reported by Aydin et al. [31] were 76 pg/mL (LOD) and 92–124% (assay recovery).

#### 2.6.2. Sample Preparation

No differences have been detected in HM obestatin concentrations between colostrum and mature milk [31]. No data were available to compare the differences between whole and skim milk concentrations of obestatin.

#### 2.6.3. Maternal Influence on HM Obestatin

The relationship between maternal BMI and HM obestatin was studied in 31 lactating women with a wide BMI range (21–36 kg/m^2^), with no association found [31]. However, Savino et al. observed a positive correlation between obestatin concentrations in HM and maternal serum [92].

#### 2.6.4. HM Obestatin and Infant Outcomes

To the best of our knowledge, data that investigate HM obestatin in relation to infant anthropometrics or body composition have not been reported in the literature.

### 2.7. Apelin

Apelin is a small peptide that results from a cleavage of pre-proprotein of 77 amino acids into a signal peptide from the N-terminal region and 55 amino acids apelin precursor called proprotein. The proprotein precursor produces a group of active peptides that includes apelin-36, apelin-17, apelin-13 and apelin-12 [124,125]. Apelin peptides are endogenous ligands of the apelin receptor (APJ), this APJ is a class A G-protein-coupled receptor that is widely distributed in several tissues such as the heart, lung, brain, kidney, adipose tissue, vascular epithelium, gastrointestinal tract and mammary gland [126]. Habata et al. [127] reported the highest expression of apelin mRNA in the mammary gland of pregnant rats, and this biologically active apelin and its mRNA increased substantially during pregnancy and lactation [127]. Immunohistochemistry techniques have detected apelin immunoreactivity in the ductal and lobular epithelial cells and vascular endothelial cells of human mammary gland [128]. Apelin has been demonstrated to be involved in the regulation of cardiovascular and fluid homeostasis, food intake, cell proliferation, angiogenesis and most importantly, glucose and lipid metabolism. Apelin also appears to possess anti-obesity and anti-diabetic properties [129].

#### 2.7.1. Analytical Methods

The only study that has measured apelin-13 and apelin-36 in HM used an enzyme immunoassay (EIA) [102], however, no performance parameters were reported (Table 7).

#### 2.7.2. Sample Preparation

Apelin was analysed in the single study that used whole milk, however no details of sample preparation were reported [102].

#### 2.7.3. Maternal Influence on HM Apelin

The reported concentrations of apelin-36 and apelin-12 in colostrum are 4.9 ± 2 ng/mL and 4.3 ± 1.2 ng/mL, respectively. The concentrations of mature milk apelin-36 and apelin-12 are 6.2 ± 1.9 ng/mL and 5.4 ± 1.8 ng/mL, respectively. Positive correlations between concentrations in colostrum and mature milk and between mature milk and maternal serum have been demonstrated. Moreover, milk from mothers with GDM has reportedly significantly lower concentrations of apelin compared to the milk of mothers without GDM [102].

#### 2.7.4. HM Apelin and Infant Outcomes

Data that describe the potential associations between HM apelin and infant growth parameters are absent.

### 2.8. Preanalytical Considerations

The published data of HM metabolic hormones are highly complex, and interpretation is problematic due to heterogeneity of the study designs, and sample collection, preparation and analysis. Therefore, when designing HM metabolic hormone studies, selection of an appropriate HM sampling protocol is just as important as the use of an appropriate analysis method.

#### 2.8.1. HM Sampling for Hormones Analysis

Sampling for HM assays of hormones is influenced by the aim of the analysis and previous knowledge with respect to changes in concentration over the course of a breastfeed, over 24 h and across lactation [93,120]. However, the majority of HM hormone analysis studies do not consider or specify the timing of samples collection, i.e., pre-, mid- or post-breastfeed, often accepting random samples from unspecified time points. Breastfed infants feed on demand yet little is known about the development of the infant appetite control mechanism, which in itself is a major regulator of energy intake. A pre- and post-feed sampling protocol would be an advantage in investigation of whether HM hormone concentrations act as a satiety signal. Indeed, changes in concentrations between pre- and post-feed samples have been studied in some HM hormones. For example, the HM leptin concentration was not different between pre- and post-feed samples [38,104], whilst ghrelin concentration was lower in pre-feed in comparison to the post-feed samples [104].

Mid-feed sampling is another common practice; however, this method limits the ability to precisely measure the changes in analyte concentrations during a feed [107]. Further, timing of the sampling is challenging, as it is not possible to determine the middle of the feed/expression. Another sampling protocol involves collecting an aliquot from a volume of breast expression [58], conducted either manually or with a breast pump. This protocol is relevant to establish the concentration of the volume removed rather than in pre- and post-feed samples. Whilst the effect of the expression method (breast pump versus the manual expression) has not been investigated in regard to concentrations of multiple components in HM, a study of HM macronutrients indicated that both methods could be used interchangeably [130], however, the volume of milk removed should be considered to avoid the erroneous conclusions for HM components that change in concentration throughout a feed, such as fat or lipophilic components.

Sampling with regards to the time of day is another pre-analytical consideration, particularly when HM and maternal serum hormone concentrations are related and thus may display circadian variation. Collecting samples in the morning is a common sampling practice, and sometimes involves maternal fasting overnight [31,83,96]. Alternatively, samples might be collected in the morning and/or 1.5 h after the first breastfeed, as well as after 1.5–2 h of maternal fasting as this practice suggested as a control for diurnal variation [63].

Recent literature indicates that sampling over a 24-h period is a growing practice. Several HM components display a circadian variation potentially playing a role in the regulation of infant growth and development with regard to their biological clock [131,132,133]. Among them are hormones such as melatonin, cortisol and prolactin [133], as well as skim milk leptin that reportedly increases at night, indicating a possible 24-h pattern [44]. Typically, samples are collected pre- and post-feed over 24 h, and then either analysed at each time point or pooled together to represent the content for the 24 h on the day of collection [44,47,56,111]. This practice might be complicated by maternal conditions, such as low milk supply or inability to collect samples throughout the 24 h.

To establish the robust sampling methods for HM hormones analysis, it is essential to consider all previously mentioned sampling protocols. Adding test-weighing of the infant before and after each breastfeed in a 24 h period to sample collection will give an opportunity to measure actual milk intake [134] and calculate infant’s daily intakes of the hormones, thus enabling evaluation of the dose-effect of the component rather than association of HM hormone concentrations with infant outcomes [7]. Thus, calculated daily intake may be a more relevant factor than concentrations when examining the nutritional physiology of the breastfed infant.

#### 2.8.2. HM Storage for Hormone Analysis

As with analysis of hormones in all biological samples, care must be taken to minimise the degradation of HM peptide hormones. While immediate hormone analysis is desirable to avoid any compositional changes caused by protease activity, this is not normally practical in HM research, thus proper sample storage and preservation is essential. Inadequate storage and preservation may impact the reproducibility and interpretation of results.

Common practice of freezing the samples at temperatures such as −20 °C, −70 °C or −80 °C has been regarded as an effective method to maintain the integrity of the HM peptides [135]. The effect of storage on HM adiponectin, leptin, resistin and ghrelin reportedly results in less than 5% loss in concentration when stored at −20 °C for a period of 1–3 months [82]. These limited data on the impact of storage condition (time and temperature) on metabolic hormone profiles of HM require further investigation considering HM as a matrix that has different physico-chemical properties in comparison to other body fluids such as blood, urine and cerebrospinal fluid [136].

In addition, the effect of freeze-thaw cycles is another consideration for HM hormone measurement. A study of HM insulin reported that it is stable at room temperature for a maximum of 12 h, at 4 °C for at least 72 h, at −20 °C for at least 2.5 years, and during at least five freeze-thaw cycles [116]. These outcomes give confidence to transferring HM samples on ice until storage at −20 °C or lower is available. However, further investigation is mandatory to improve this area of HM composition research.

Although there are protease inhibitors in HM, their concentrations may be low and vary at different stages of lactation [137], thus the use of exogenous protease inhibitors at the time of collection might preserve proteins and peptides of interest [138]. Protease inhibitors are proposed for HM hormone studies that investigate HM ghrelin. The use of aprotinin as a protease inhibitor and 1N HCl as an acidification agent has improved measurement of HM acylated-ghrelin (aGh) by protecting aGh from proteolytic activity [108]. However, there is no HM hormone studies that have investigated the effect of adding exogenous protease inhibitors on the concentration of other peptide hormones.

### 2.9. Analytical Considerations and Methods Validation

The scope of the methods used to measure HM hormones is outlined in Table 1, Table 2, Table 3, Table 4, Table 5, Table 6 and Table 7. The methods used to quantify HM hormones can be classified as immunoassay, immunosensor and chromatography methods. Immunoassay methods are bioanalytical methods in which quantitation of analyte depends on its reaction with specific antibodies. Immunosensor is an analytical device that coupled an immunochemical reaction with a transducer that creates a measurable signal that reflects the concentration of a specific analyte. Finally, chromatography methods are based on separating, identifying and quantifying analytes in a mixture.

The methods and instruments used to measure HM hormones have made remarkable progress in the past few decades. However, critical consideration of HM hormone analysis is the ability of a kit to measure the hormone of interest in HM. Therefore, analytical method validation parameters such as linearity, recovery, precision and LOD must be tested to ensure the reliability of results, as most HM hormone analyses have been adopted from other body fluids.

Numbers of methods are available to measure HM hormones; however, immunoassay methods have been predominantly used to measure HM hormones. RIA methods have been used successfully to determine HM hormones, as it has the advantages of being precise and extremely sensitive. However, RIA is susceptible to interference with triglycerides; therefore, skimming milk is essential to achieve the optimum performance of the assay. Still, skimming milk is a time-consuming step, which negatively influences HM hormone concentrations (e.g., leptin). Thus, the concentration of HM hormones in skim milk might not represent the actual amount of HM hormones consumed by infants [48]. As a result, HM ELISA assay has been successfully optimised to overcome the limitation of RIA and measure hormones in whole HM [28,47,48,58,111]. These findings highlight the importance of optimising hormone measurement in whole HM to accurately examine the amount of the hormone received by the infant during a breastfeed. Therefore, optimising HM assay methods to measure hormones in whole HM after homogenising the sample using suitable homogenisation techniques, if possible, and considering analytical method interference when selecting an analytical technique is recommended.

### 2.10. Human Milk Hormones and Infant Outcomes

An epidemic of childhood obesity and non-communicable diseases associated with it are of increasing international concern. The lifelong risk of non-communicable diseases could be modified through early nutrition and other environmental factors during the period of conception to early childhood [139]. Breastfed infants display a variety of feeding patterns and milk intakes throughout the day, self-regulating their energy intake [134]; however, why there is such variation is unclear, with factors such as HM composition as well as foetal exposure and infant genetics all likely to play a role. Non-nutritive bioactive components of HM, such as hormones, may enter into infant’s circulation via several potential mechanisms, which have been previously described [140,141], and may influence infant development and health outcomes, contributing to the development of infant body composition and appetite control, regulation of infant food intake, weight and adiposity, and prevention of obesity [142].

Indeed, in infants, HM is believed to be a major source of hormones, such as leptin, with the endogenous leptin-synthesising mechanisms being still immature [143], ensuring higher serum levels of leptin in breastfed infants compared to those consuming formula [144]. With all mucosae in human foetus starting to express the leptin Ob-Rb receptor between the 7th and 9th week of gestation, the important role of leptin in the developmental process and maturation of the human gastrointestinal tract [145] makes it biologically plausible for the hormones ingested by the infant to be absorbed and participate in regulation of infant appetite and metabolism.

Multiple studies report, predominantly the effect of leptin and adiponectin, on infant growth and more recently body composition. However, most studies have focused on relationships of hormone concentrations rather than infant daily intakes, thus warranting careful consideration when interpreting the results (a brief summary is presented in Tables S1–S7). Nevertheless, the first step in ensuring the correct determination of the daily intake is an accurate concentration measured in optimally collected HM sample.

## 3. Materials and Methods

This review is not intended to be a systematic review, however we adhered to the systematic review guidelines where appropriate. The literature search was conducted using MEDLINE. The search was limited to human studies with no date range restrictions. The keywords terms and medical subject headings (MeSH) included (breast milk OR human milk OR milk, human) AND (Leptin OR Insulin OR Adiponectin OR Ghrelin OR Resistin OR Obestatin OR Apelin). The primary reviewer (MS) removed duplication using Endnote. The proposed literature search was conducted between 20 April and 10 June 2020, and the monthly search alert of the database was set up to ensure literature coverage with the last search conducted in May 2021. Case reports, commentaries, letters to the editor, and reviews were not included in the review, as well as papers written in languages other than English. The reference list of related titles and abstracts was scanned, and data were extracted and summarised in a table format, with no manipulation to the results or units of measurement with the exception of insulin. Where possible, insulin measurement unit was converted from μIU/mL to pmol/L based on unit conversion for human insulin of 1 μIU/mL = 6.00 pmol/L so the data are comparative [146]. From the reviewed studies, associations found between HM component levels and maternal and infant anthropometry were also noted in the summary table. Any disagreements were resolved through discussion involving an additional reviewer (ZG). No data analysis was performed on the results of the studies included in this review due to studies heterogeneity. When necessary, authors of interest were contacted for additional information, unpublished results or discrepancies in the literature.

## 4. Conclusions

HM metabolic hormones are essential for the growth, development and health of the infant. Thus, it is crucial to a better understand their short- and long-term effect on infant health. Interest in HM hormones is progressively increasing. Therefore, more thorough studies are required that employ standard sampling and preparation, and validated protocols to accurately measure the hormones in order to discover the mechanism by which these hormones are modifiable by maternal factors and impact infant growth and development.

## Figures and Tables

**Table 1:** Studies examining human milk leptin.

Author	Year	Sample Size (n)	Sample Type	Sampling	Time of the Day (h)	Time Postpartum	Preparation	Method	Intra, Inter Assay (%CV)	LOD (ng/mL)	R (%)	Concentration (ng/mL)
Houseknecht et al. [20]	1997	14	-	12 h fast	08:00	-	SM and Wm	RIA	3.0, 12.6	0.3	SM: 90 WM: 82	WM, 10.1 ± 2.6; SM, 1.5 ± 0.87
Uçar et al. [21]	2000	18	C, M	pre- and post-feed	9:00–12:00	3–120 d	SM	RIA	-	0.5	-	(0.8–15) (range)
Lönnerdal and Havel [22]	2000	-	M	-	-	2–4 mo	SM and WM	RIA	-	-	-	WM, 32.7 ± 14.1 SM, 0.2 ± 0.1
Resto et al. [23]	2001	29	M	-	-	1–4 wk	M, added pancreatic lipase, NaHCO_3_	RIA	-	-	-	5.28 (24.79)
Uysal et al. [24]	2002	50	M	2 h after last feed	8:00–10:00	1,2, 3 mo	SM	RIA	<6, -	0.1	-	Non-Ob, 0.37 ± 0.4 Ob, 0.27 ± 0.2
Bielicki et al. [25]	2004	33	C, M	1–3 h after breakfast	-	2–3, 4–5 d 4–6 wk	WM	RIA	<10, 9.7	0.25	97	Preterm, 0.63 ± 0.18 Term, 1.34 ± 0.14
Dundar et al. [26]	2005	47	M	pre-feed	10:00–11:00	15 d, 1, 2, 3 mo	WM	RIA	6.2, -	0.5	83 ± 4	15 d, AGA, 18.4 ± 2; LGA, 28.5 ± 4.4; SGA, 13.4 ± 2.2
Ilcol et al. [27]	2006	160	C, M	pre-feed, 3 h after breakfast	morning	2, 8, 25 d	WM	IRMA	<5, 7	0.1	-	2 d, 3.35 ± 0.25; 8 d, 2.65 ± 0.21; 25 d, 1.63 ± 0.18
Bronsky et al. [28]	2006	59	C	collected on EDTA and aprotinin	after 7:00	1–2 d	SM	ELISA	7.6, 9.1	0.05	-	0.50 (0.05) mean (SEM)
Miralles et al. [29]	2006	28	M	post-feed	morning	1, 3, 6, 9 mo	WM	ELISA	-	0.008	103.1 ± 1.4	1 mo, 0.156 ± 0.039
Weyermann et al. [17]	2006	766	M	pre-feed	-	6 wk, 6 mo	SM	ELISA	<7	-	-	12.8 ± 10.1
Weyermann et al. [30]	2007	767	M	-	-	6 wk	SM	ELISA	<7, -	-	-	0.175 (0–4.1) median (range)
Aydin et al. [31]	2008	31	C, M	pre-feed, collected on aprotinin	9:00	2, 25 d	SM	ELISA	-	-	-	C, 2.01 ± 0.34; M, 2.04 ± 0.67
Savino et al. [32]	2010	36	-	-	-	<6 mo	SM	RIA	<5, 8	0.04	-	0.51 (0.34)
Bronsky et al. [33]	2011	72	C, M	collected on EDTA + protease inhibitor	-	1 d; 1, 3, 6, 12 mo	SM	ELISA	6, 9	0.5	-	1 d, 0.3 ± 0.04; 1 mo, 0.2 ± 0.03; 3 mo, 0.1 ± 0.01; 6 mo, 0.1 ± 0.02; 12 mo, 0.2 ± 0.04
Schuster et al. [34]	2011	23	C, M	-	-	1, 2, 3, 4 wk 2, 3, 4, 5, 6 mo	SM	RIA	-	0.01	70.2 ± 8.7	1 wk, 0.21 ± 0.19 6 mo, 0.18 ± 0.15
Eilers et al. [35]	2011	77	C, M	fasting 2 h prior sampling	16:00–20:00	3 d, 28 d	SM	RIA	5, 6	-	~94	Preterm, 3 d, 0.7 ± 0.79; 28 d, 0.5 ± 0.4; Term, 3 d, 0.65 ± 0.67; 28 d, 0.5 ± 0.4
Fields and Demerath [36]	2012	19	M	complete breast expression	8:00–10:00	~1 mo	SM	-	-	-	-	0.95 ± 0.73
Savino et al. [37]	2012	23	-	-	-	<6 mo	SM	ELISA	<5, 6.8	6	-	2.34 (5.73)
Schueler et al. [38]	2013	13	M	overnight fast, pre- and post-feed	7:00–10:00	29–38 d.	SM	RIA	9.4, -	-	-	pre, 0.9 ± 0.7 post, 1.0 ± 0.8
Chang et al. [39]	2013	-	-	-	-	3–4 mo	SM	ELISA	<5, 6	0.5	-	-
Ojeda et al. [40]	2013	-	-	-	-	2 mo	-	ECIs, & ELISA			+	ECIs, 2.6 ± 0.1 ELISA, 2.9 ± 0.5
Kon et al. [41]	2014	103	M	-	Morning	1, 2, 3 mo	SM	ELISA	3.7, -	0.42	-	LWG, 1 mo, 1.63 (0.27); 3 mo, 1.35 (0.31); NWG, 1 mo, 1.55 (0.17); 2 mo, 1.83 (0.23); 3 mo, 3.29 (0.70) HWG,1 mo: 1.53 (0.29); 2 mo, 2.20 (0.28); 3 mo, 3.57 (1.37) mean (SEM)
Brunner et al. [42]	2014	6 wk, 152 6 mo, 120	M	overnight fast	Morning	6 wk, 6 mo	SM	RIA	-	-	-	6 wk, 0.11 (0.19); 6 mo, 0.09 (0.18)
Khodabakhshi et al. [43]	2015	Ob, 40 NW, 40	M	overnight fast, 2 h after last breast feed	8:00–10:00	2–5 mo	SM	ELISA	-	-	-	NW, 1.81 (1.65–1.94) Ob, 1.78 (1.67–1.94)
Cannon et al. [44]	2015	19	M	pre- and post-feed	24 h milk collection	3–21 wk	SM	RIA	-, 9.9	0.017	98.4 ± 6.8	pre, 0.43 ± 0.10, post, 0.42 ± 0.11
Quinn et al. [45]	2015	113		mid-feed sampling	6:00–10:00	10 d–36 mo	SM	ELISA	-	-	-	0.3 ± 0.29
Andreas et al. [46]	2016	120	-	1 h fast prior sampling, pre- and post-feed, on protease inhibitors	10:00–13:00	1 wk, 3 mo	SM	Multiplex assay	3, -	3.1	-	1 wk, pre 541.7 (161.2); post 614.4 (172.1) 3 mo, pre 684.8 (117.4); post 464.3 (125.1) mean (SEM)
Gridneva et al. [47]	2016	27	M	pre- and post-feed	09:30–11:30	2–5 mo	SM and WM	ELISA	-, <7.2	-	97.7 ± 9.7	WM, 0.51 ± 0.18 [0.23–1.10] SM, 0.28 ± 0.12 [0.20–0.84]
Kugananthan et al. [48]	2016	61	M	pre- and post-feed	11:00	2, 5, 9, 12 mo	SM and WM	ELISA	<5, 7.2	-	97.7 ± 9.7	WM, 2 mo, 0.50 ± 0.16; 5 mo, 0.48 ± 0.16; 9 mo, 0.56 ± 0.11; 12 mo, 0.54 ± 0.14 SM, 2 mo, 0.32 ± 0.16; 5 mo, 0.26 ± 0.07; 9 mo, 0.22 ± 0.03; 12 mo, 0.21 ± 0.02
De Luca et al. [49]	2016	100	M	over-feed	9:00–11:00	1 mo	SM	RIA	<10, -	0.5	+	Non-Ob, 2.5 [2.1–3.0] Ob, 4.8 [4.1–5.6] mean (95% CI)
Savino et al. [50]	2016	58	-	collected on protease inhibitor	7:00–9:00	-	SM	RIA	-	0.04	-	0.89 (1.32)
Fields et al. [51]	2017	37	M	complete breast expression	8:00–10:00	1, 6 mo	SM	-	-	-	-	1 mo (n = 37), 0.589 (0.353–1.45) 6 mo (n = 30), 0.426 (0.145–940)
Meyer et al. [52]	2017	147	M	overnight fast	-	6 wk, 4 mo	-	RIA	-	-	-	-
Quinn and Childs [53]	2017	116	-	mid-feed	6:00–10:00	-	SM	ELISA	17.8, -	-	-	0.27 ± 0.25
Nunes et al. [54]	2017	69	C, M	-	-	1, 2, 30 d	SM	ELISA	-	-	-	SGA, M, 0.377 (0.36–0.41) GDM, M, 0.460 (0.45–0.70) CTL, M, 0.715 (0.48–0.9)
Cannon et al. [55]	2017	20	-	pre- and post-feed	24 h milk collection	6–32 wk	SM	ELISA	-, 9.9	0.017	98.4 ± 6.8	0.51 ± 0.16 (0.42–1.15)
Kugananthan et al. [18]	2017	59	M	pre- and post-feed	9:30–11:30	2, 5, 9, 12 mo	SM and WM	ELISA	-, <7.2	0.05	SM, 96.3 ± 1.2 WM, 97.1 ± 9.1	WM, 0.53 ± 0.19 (0.20–2.24) 2 mo, 0.55 ± 0.29; 5 mo, 050 ± 0.17; 9 mo, 0.53 ± 0.15; 12 mo, 0.54 ± 0.13 SM, 0.28 ± 0.12 (0.19–1.46) 2 mo, 0.34 ± 0.21; 5 mo, 0.27 ± 0.07; 9 mo, 0.26 ± 0.09; 12 mo, 0.26 ± 0.08
Gridneva et al. [7]	2018	20	M	pre- and post-fed	24 h milk collection	2, 5, 9, 12 mo	SM and WM	ELISA	-, <7.2	0.05	97.1 ± 9.1	WM, 2 mo, 0.50 ± 0.18; 5 mo, 0.49 ± 0.17; 9 mo, 0.56 ± 0.11; 12 mo, 0.50 ± 0.11 SM, 2 mo, 0.34 ± 0.20; 5 mo, 0.26 ± 0.08; 9 mo, 0.21 ± 0.02; 12 mo, 0.21 ± 0.03
Chan et al. [56]	2018	430	M	pooled 24 h milk collection	24 h milk collection	4 mo	SM	ELISA	-	0.137	_	349 (186–689)
Yu et al. [57]	2018	96	C, M	pre- and post-feed for mature milk	3 d, 8:00–9:00 42, 90 d, 14:00–16:00	3, 42, 90 d	SM	ELISA	<7.4, 9.3	-	-	GDM (n = 48), 3 d, 1.28 (0.87–2.63); Healthy (n = 48), 3 d, 1.49 (0.56–3.25)
Sadr Dadres et al. [58]	2019	135	M	2 h after feeding	10:00–12:00	1, 3 mo	SM	ELISA	<6, 6	0.008	90–94	1 mo, 0.640 ± 0.606 3 mo, 0.484 ± 0.672
Zamanillo et al. [59]	2019	59	M	-	9:00–14:00	30, 60, 90 d	WM	ELISA	-	-	-	1 mo, 0.376 ± 0.353; 2 mo, 0.341 ± 0.314; 3 mo, 0.346 ± 0.333
Logan et al. [60]	2019	SPATZ, 1090 UBCS, 1006	M	1 h after last feed	9:00–12:00	6 wk, 6 mo	SM	ELISA	<7.0, -	-	-	SPATZ, 6 wk, 266.5 (152.0–498.0); UBCS, 6 wk, 175.0 (79.8–350.0)
Larrosa Haro et al. [61]	2019	131	M	pre- and post-feed	-	8, 16 wk	SM	ELISA	-	0.01	-	8 wk, pre, 0.316 (0.50); post, 0.317 (0.57); 16 wk, pre, 0.447 (0.48); post, 0.411 (0.64)
Logan et al. [62]	2019	694	M	1 h after last feed	9:00–12:00	6 wk, 6 mo, 12 mo	SM	ELISA	-, 5.8	-	-	6 wk (n = 668), 388.8 ± 398.1; 6 mo (n = 445), 269.6 ± 305.3; 12 mo (n = 69), 320.4 ± 345.2
Kocaadam et al. [63]	2019	65	M	pre-feed, ~2 h fast prior, ~2 h after last feed, on EDTA and aprotinin	8:00–11:00	15–30 d	SM	ELISA	-	-	-	preterm, (n = 31), 2.0 (2.5) term, (n = 34), 0.0 (2.3)
Schneider-Worthington et al. [64]	2020	25	M	-	-	1 mo	SM	ELISA	4.48, 13.56	-	-	0.47 (0.94)
Dal Bello et al. [65]	2020	-	-	-	-	-	-	nano-HPLC-HRMS	-	27	-	6.70
Galante et al. [66]	2020	501	-	pre-feed	10:00–24:00	2–3 mo	SM	ELISA	4, 8	-	-	0.116 ± 0.112, ng/mg (raw values corrected per mg of protein per mL)
Joung et al. [67]	2021	50	C, M	post-feed, 24 h pooled	-	7, 14, 21 and 28 d	WM	ELISA	10	0.01	-	-

Data reported as mean ± SD or median (IQR) unless specified. AGA, appropriate age for gestational age; C, colostrum milk; CTL, control group; CI, confidence interval; CV, coefficient variation; d, day; ECIs, electrochemical immunosensor; EDTA, ethylenediaminetetraacetic acid; ELISA, enzyme-linked immunosorbent assay; GDM, gestational diabetes mellitus; IRMA, immunoradiometric assay; LGA, large for gestational age; LOD, limit of detection; M, mature milk; mo, month; Non-Ob, without obesity; Ob, with obesity; R, recovery of analyte; RIA, radioimmunoassay; SGA, small for gestational age; SM, skim milk; SPATZ, Ulm SPARTZ health study; UBCS, Ulm birth cohort study; wk, week; WM, whole milk; y, year.

**Table 2:** Studies examining human milk adiponectin.

Author	Year	Sample Size (n)	Sample Type	Sampling	Time of the Day (h)	Time of Collection	Preparation	Method	Intra, Inter Assay (%CV)	LOD (ng/mL)	R (%)	Concentration (ng/mL)
Bronsky et al. [28]	2006	59	C	collected on EDTA and aprotinin	7:00	1–2 d	WM	ELISA	3.8, 5.4	0.5	91.6	13.7 (0.8) mean (SEM)
Weyermann et al. [17]	2006	766	M	pre-feed	-	6 wk, 6 mo	SM	ELISA	<7, 7	-	-	12.8 ± 10.1
Weyermann et al. [30]	2007	674	M	-	-	33–71 d	SM	ELISA	<7, 7	-	-	0.175, median
Martin et al. [75]	2006	158	M	no feed 2 h before sampling	10:00–13:00	1 d–12 mo	SM	RIA	3.9, 5.8	-	109 ± 6.7	17.7 (4.2–87.9) median (range)
Woo et al. [76]	2009	322	M	-	10:00–13:00	1 wk–6 mo	SM	RIA	8.5, 3.9	-	-	24.0 ± 8.6
Dündar et al. [81]	2010	25	C	-	08:00–10:00	-	WM	ELISA	-	0.78	-	29.5 ± 6.4, (1.26–77.1) (range)
Bronsky et al. [33]	2011	72	C, M	post-feed	after 7:00	1 d, 1, 3, 6, 12 mo	WM	ELISA	3.8, 5.4	0.5	91.6	3 mo, 20.5 (0.6) 6 mo, 21.4 (0.8) 12 mo, 25.7 (1.4) mean (SEM)
Luoto et al. [79]	2011	30	C	-	-	0–3 d	-	DELFIA	<5.8	0.8	-	(3.1–98.9) (range)
Ley et al. [82]	2011	34	M	pooled milk bank samples	-	1–6 mo	SM and WM	RIA	-, 9.3	-	WM, 105.5 ± 18.2 SM, 118.4 ± 36	WH, 12.3 ± 6.1; SM, 12.1 ± 5.4
Cesur et al. [83]	2012	25	M	pre-feed	btw 8–10	1 and 4 mo	SM	ELISA	-	0.78	-	1 mo, 23.61 ± 32.95; 4 mo, 6.66 ± 9.48
Luoto et al. [80]	2012	256	C	-	-	0–3 d	-	DELFIA	<5.8	0.8	-	(2.6–317) (range)
Ley et al. [78]	2012	170	C, M	no feed 2 h before sampling	-	2 d and 3 mo	SM	RIA	-, 9.3	-	118.4 ± 36	C, 50.0 (21.9, 104.6); M, 12.3 (9.9, 17.2) median (25th, 75th percentile)
Liu et al. [84]	2012	48	C	-	-	3 d	SM	ELISA	-	-	-	PE, 118.1 ± 21.6; Healthy, 59.9 ± 10.7
Ozarda et al. [77]	2012	157	C, M	pre-feed, 3 h after breakfast	morning	1–180 d	SM	RIA	<6, 8	-	-	34.8 ± 2.4, (4.0–105.7) (range)
Savino et al. [85]	2012	60	-	-	7:00–8:00	<6 mo	SM	ELISA	<4.7, 6,7	<0.6	-	9.99 (3.59, 20.52), median (25th, 75th percentile)
Woo et al. [86]	2012	277	-	-	10:00–13:00	1 wk, and 1, 2, 5, 6 mo	SM	RIA	-	-	-	21.57 ± 5.12
Kon et al. [41]	2014	103	M	-	morning	1, 2 and 3 mo	SM	ELISA	5.9, -	26	-	LWG, 1 mo, 1.06 (0.10); 3 mo, 1.09(0.15); NWG, 1 mo, 1.14 (0.09); 2 mo, 1.04 (0.09); 3 mo, 1.14 (0.08); HWG, 1 mo, 1.10 (0.09); 2 mo, 1.15 (0.08); 3 mo, 1.12 (0.10); mean (SEM); µg/mL
Brunner et al. [42]	2014	6 wk, 152 6 mo, 120	M	overnight fast	morning	6 wk 6 mo	SM	RIA	-	-	-	6 wk, 10.93 (8.34); 6 mo, 10.36 (9.40)
Khodabakhshi et al. [43]	2015	Ob, 40 NW, 40	M	2 h after feed, fasting	8:00–10:00	2–5 mo	SM	ELISA	-	-	-	NW, 330.05 (298.33–376.81) Ob, 323.48 (281.14–350.89)
Gridneva et al. [47]	2016	27	M	pre- and post-feed	09:30–11:30	2–5 mo	WM	ELISA	-, <7.2	1	96.2 ± 3.2	10.02 ± 4.08 (6.18–22.58) (range)
Anderson et al. [87]	2016	117	M	no feed 2 h prior sampling	6:00–10:00	9 d–24 mo	SM	ELISA	<10, 14	-	-	7.47 ± 5.75 (1.38–19.1) (range)
Nunes et al. [54]	2017	69	C, M	-	-	1, 2, 30 d	SM	ELISA	-	-	-	SGA, M, 9.99 (5.16–19.21); GDM, M, 12.43 (6.9–14.87); CTL, M, 9.87 (6.33–11.5)
Quinn and Childs [53]	2017	116	-	mid-feed sampling	6:00–10:00	-	SM	ELISA	<14.4, -	-	-	4.1 ± 2.13
Kugananthan et al. [18]	2017	59	M	pre- and post-feed	9:30–11:30	2, 5, 9, 12 mo	WM	ELISA	-, <2.5	1	96.2 ± 3.2	2 mo, 11.12 ± 4.39; 5 mo, 9.30 ± 3.94; 9 mo, 8.46 ± 2.26; 12 mo, 11.07 ± 7.88 9.88 ± 5.05, (4.56–54.92) (range)
Gridneva et al. [7]	2018	20	M	pre- and post-feed	24 h milk collection	2, 5, 9, 12 mo	WM	ELISA	-, <2.5	1	96.2 ± 3.2	2 mo, 11.14 ± 5.79; 5 mo, 8.42 ± 1.69; 9 mo, 8.44 ± 1.33; 12 mo, 11.22 ± 4.22 (5.66–22.88) (range)
Chan et al. [56]	2018	430	M	pooled 24 h milk collection	24 h milk collection	4 mo	SM	ELISA	-	0.064	-	19.5 (14.5–25.9)
Yu et al. [57]	2018	96	C, M	pre- and post-feed for mature milk	8:00–9:00, d 3; 14:00–16:00, d 42 and 90	3, 42, 90 d	SM	ELISA	<5.4, 8.5	-	-	GDM (n = 48), 3 d, 21.7 (14.7–56.1); 42 d, 11.9 (8–18·37); 90 d, 11 (7 8.5–13.9); Healthy (n = 48), 3 d, 65.8 (29.8–126.9); 42 d, 12.22 (9.7–14.9); 90 d, 15.3 (11.6–19.5)
Young et al. [88]	2018	41	M	mid-feed	morning	2 wk, 1, 2, 3 and 4 mo	SM	RIA	-	-	-	2 wk, 19.4 ± 9.0; 1 mo, 19.2 ± 11.2; 2 mo, 17.3 ± 12.5; 3 mo, 20.9 ± 27.6; 4 mo, 15.0 ± 7.9
Mohamad et al. [89]	2018	155	C, M	pre- and post-feed	-	1 d, 2 mo	SM	ELISA	<10, 10	-	-	1 d, 17.05 ± 8.75 2 mo, 11.53 ± 8.45
Sadr Dadres et al. [58]	2019	135	M	2 h after last feed	10:00–12:00	1 and 3 mo	SM	ELISA	<6, 6	1	98–120	1 mo, 16.8 ± 9.6 3 mo, 15.6 ± 15.2
Zamanillo et al. [59]	2019	59	M	-	9:00–14:00	30, 60 and 90 d	WM	ELISA	-	-	-	1 mo, 23.39 ± 7.57; 2 mo, 20.83 ± 6.61; 3 mo, 20.32 ± 8.02
Grunewald et al. [90]	2019	367	M	-	-	16 d, 163 d	SM	ELISA	4.5, 4.8	-	91.6 ± 4.0	19.28 ± 6.63
Kocaadam et al. [63]	2019	65	M	pre-feed, ~2 h fast prior, ~2 h after last feed, on EDTA and aprotinin	8:00–11:00	15–30 d	SM	ELISA	-	-	-	Pre-term, 24.6 (14.3); Term, 22.9 (9.7)
Schneider-Worthington et al. [64]	2020	25	M	-	-	1 mo	SM	ELISA	4.48, 13.56	-	-	14.90 (9.19)
Galante et al. [66]	2020	501	M	pre-feed	10:00–24:00	2–3 mo	SM	ELISA		-	-	0.404 ± 0.237, ng/mg (raw values corrected per mg of protein per mL)
Joung et al. [67]	2021	50	C, M	post-feed, 24 h pooled, samples pre-treated with protease containing buffer	-	7, 14, 21 and 28 d	WM	ELISA	<6	0.01	-	-

Data reported as mean ± SD or median (IQR) unless specified. C, colostrum milk; CTL, control; CV %, coefficient variation percentage; d, day; DELFIA, dissociation-enhanced lanthanide fluorescence immunoassay; EDTA, ethylenediaminetetraacetic acid; ELISA; enzyme-linked immunosorbent assay; HWG, high weight gain; LOD, limit of detection; M, mature milk; mo, month; NW, with normal weight, NWG, with normal weight gain; Ob, with obesity; PE, women with preeclampsia; R%, recovery of analyte percentage; RIA: radioimmunoassay; SGA, small for gestational age; SM, skim milk; WM, whole milk; wk, week.

**Table 3:** Studies examining human milk ghrelin.

Author	Year	Sample Size (n)	Sample Type	Sampling	Time of the Day (h)	Time of Collection	Preparation	Method	Intra, Inter Assay (%CV)	LOD (pg/mL)	R (%)	Concentration (pg/mL)
Aydin et al. [96]	2006	17	C, T, M	overnight fasting	morning, before breakfast	1, 7, 15 d	SM	RIA, tGh	<7.2, 11	12.5	105 ± 7	C, 70.3 ± 18; T, 83.8 ± 18; M, 97.3 ± 13
Kierson et al. [98]	2006	10	M	-	-	7–21 d	SM and WM	RIA, tGh	-	-	-	WM, 2125 (260–6000) SM, 595 (420–1300) median (range)
Aydin et al. [99]	2007	29	C and M	overnight fasting, aprotinin added to sample analysis with HPLC	morning, before breakfast	2, 15 d	SM	RIA and HPLC	<10, 14	RIA, ~9 HPLC, ~15	RIA, 92 HPLC, -	RIA, GDM, 26.1 ± 7.4; non-GDM, 64 ± 9.1; HPLC, dGh, (412–788); aGh, (15–30); tGh, (427–818) (range)
Ilcol and Hizli [101]	2007	159	C, T and M	3 h after breakfast, one set acidified with 1N HCL for aGh analysis	morning	1–3, 4–14, 15–30, 30–90, 91–180 d	SM	RIA	<8, 11	100	98	C, aGh, 450 ± 25; tGh, 880 ± 80 M, aGh, 801 ± 43; tGh, 3249 ± 378
Aydin et al. [100]	2008	8	-	aprotinin added	-	-	SM	HPLC	<12, 14	11 ± 2, 14 ± 3	aGh, 91 dGh, 108	aGh, 75; dGh, 1131
Aydin et al. [31]	2008	31	C, M	pre-feed, with aprotinin	9:00	2, 25 d	SM	RIA	<8, 11	100	98	C, aGh, 61.58 ± 7.11; dGh, 554.11 ± 79.42; tGh, 615.70 ± 78.39 M, aGh, 76.73 ± 10.58; dGh, 1238.5 ± 181.2; tGh, 1315.2 ± 183.9
Aydin [102]	2010	20	C, M	-	-	-		ELISA	-	-	-	C, aGh, 39.2 ± 2; dGh, 466.1 ± 52 M, aGh, 48.2 ± 5.1; dGh, 505.1 ± 52
Dündar et al. [81]	2010	25	C	-	8:00–10:00	-	WM	RIA	-	93	-	dGh, 1280 ± 32.6; tGh, 4181 ± 456
Yis et al. [103]	2010	47	M	3 h fasting	10:00–12:00	3–4 mo		EIA	<5, 14	80	-	tGh, 800 ± 500, (200–2500) (range)
Karatas et al. [104]	2011	46	M	2 h fasting, pre- and post-feed, aprotinin + 1N HCL	9:00–10:00	1–3 mo, and 4–6 mo	SM	RIA	-	-	-	tGh, pre, 288.9 ± 63.3; post, 198.72 (98.5–265.5); aGh, pre, 11.87 ± 2.54; post, 8.47 ± 1.60 median (min–max)
Savino et al. [105]	2011	20	M	before breakfast	9:00	1–5 mo		RIA	5, 7.6	93	-	828 ± 323
Cesur et al. [83]	2012	25	M	pre-feed, acidified by 1N HCL	8:00–10:00	1 and 4 mo	SM	RIA	-	tGh, 93; aGH, 7.8	-	tGh, 1 mo, 3095.18 ± 1507.25 tGh, 4 mo, 2876.75 ± 1626.35 aGh, 1 mo, 1042.97 ± 147.74 aGh, 4 mo, 1659.59 ± 155.61
Savino et al. [92]	2012	40	M	-	8:00	2–3 mo	SM	RIA	5, 7.6	93	-	526.4 (439.86)
Kon et al. [41]	2014	103	M	-	morning	1, 2, 3 mo	SM	ELISA	0.02, -	20	-	LWG, 1 mo, 0.77 (0.22); 3 mo, 8.24 (4.76) NWG, 1 mo, 7.52 (2.63); 2 mo, 5.06 (2.49); 3 mo, 0.71 (0.19) HWG, 1 mo, 2.32 (1.19); 2 mo, 6.52 (1.87); 3 mo, 3.05 (1.9); ng/mL
Khodabakhshi et al. [43]	2015	Ob, 40 NW, 40	M	2 h after feed, fasting	08:00–10:00	2–5 mo	SM	ELISA, tGh	-	-	-	Ob, 132 (130.75–136.25) NW, 137.5 (133–156)
Andreas et al. [46]	2016	120	T, M	no feed 1 h prior sampling, pre- and post-feed, protease inhibitor	10:00–13:00	1 wk and 3 mo	SM	Multiplex assay (Bio-Rad)	4, -	1.2	-	1 wk, pre-feed, 123 (15.8); post-feed, 95.8 (16.9); 3 mo, pre-feed, 101.1 (11.5), post-feed, 59.3 (12.3); ng/mL
Slupecka-Ziemilska et al. [106]	2017	40	C	2 h after meal, EDTA and aprotinin	10:00–11:00	3 d	SM	RIA	6, -	84.7	90	-
Young et al. [107]	2017	48	M	mid-feed	-	2 wk, and 1, 2, 3, 4 mo	SM	-	-	-	-	2 wk, 510 ± 4111 2 mo, 268 ± 348 4 mo, 251 ± 283
Yu et al. [57]	2018	96	C, M	pre- and post-feed, mature milk	btw 8–9 on 3 d; btw 14–16 on 42 and 90 d	3, 42, 90 d	SM	ELISA	<1.9, 7.7	-	-	GDM (n = 48), 3 d, 124·43 (89.87–178.76); 42 d, 338.74 (189.98–432.95); 90 d, 104.62 (72.72–154.71); Healthy (n = 48), 3 d, 159.36 (122.62–234.33); 42 d, 337.60 (149.82–565.77); 90 d, 210.91 (147.25–381.88)
Larrosa Haro et al. [61]	2019	131	M	pre- and post-feed	-	8 and 16 wk	SM	ELISA	-	30	-	8 wk, pre, 154.1 (75.1); post, 178.8 (88.1) 16 wk, pre, 162.4 (102.1); post, 210.6 (153.1)

Data reported as mean ± SD or median (IQR) unless specified. aGh, acylated-ghrelin; btw, between; C, colostrum milk; % CV, coefficient variation percentage; d, day; dGh, deacylated-ghrelin; ELISA, enzyme-linked immunosorbent assay; GDM, gestational diabetes mellitus; Gh, ghrelin; HCL, hydrochloric acid; HPLC, high pressure liquid chromatography; LOD, limit of detection; M, mature milk; mo, month; NW, normal weight; R%, recovery of analyte percentage; RIA, radioimmunoassay; T, transitional milk; tGh, total ghrelin; wk, week.

**Table 4:** Studies examining human milk insulin.

Author	Year	Sample Size (n)	Sample Type	Sampling	Time of the Day (h)	Time of Collection	Preparation	Method	Intra, Inter Assay (%CV)	LOD (pmol/L)	R (%)	Concentration (pmol/L)
Shehadeh et al. [114]	2003	90	C	-	-	3, 10 d	SM	RIA	6.6, -	-	-	d 3: 300.6 ± 207.6, (42–1074) d 10: 241.2 ± 208.8 (72–1098) (range)
Ley et al. [82]	2011	34	M	pooled milk bank samples	-	1–6 mo	SM	ECLIA	-	-	-	162 ± 64.2
Ley et al. [78]	2012	170	C, M	-	-	2 d, 3 mo	SM	ECLIA	-	-	-	C, 170 (64,402); M, 52 (34,87) median (25th,75th percentile)
Fields and Demerath [36]	2012	19	M	pre-, mid- and post feed (pooled), 1.5 h after the last feed	8–10	1 mo	SM	-	-	-	-	954.9 ± 726.5 pg/mL
Whitmore et al. [111]	2012	14	M	pre- and post-feed	24 h milk collection	1–6 mo	SM	ELISA & CMIA	-, < 9	CMIA, 9 ELISA, 0.8 Total ELISA, 34.2	CIMA, 100 ± 5 ELISA, 95 ± 4 Total ELISA, 97 ± 8	93.78 ± 6.18
Andreas et al. [46]	2016	120	-	no feeding 1 h prior sampling, pre- and post-feed, protease inhibitors	10:00–13:00	1 wk, 3 mo	SM	Multiplex assay	3, -	1	-	pre-feed, 1 wk, 515.4 (42.4); post-feed, 1 wk, 439.5 (45.2); pre-feed, 3 mo, 524.8 (30.9); post-feed, 3 mo, 456.8 (32.9) mean (SEM), ng/mL
Nunes et al. [54]	2017	69	C, M	-	-	1, 2, 30 d	SM	ELISA	-	-	-	SGA, M (n = 12), 109.7 (84.7–156.9); GDM, M (n = 12), 158.3 (113.2–419.4); CTL, M (21), 153.5 (92.4–223.6); median (25–75th percentile)
Young et al. [107]	2017	48	M	mid-feed	ND	2 wk, 1, 2, 3, 4 mo	SM	RIA	-	-	-	2 wk, 109.7 ± 104.2 2 mo, 136.1 ± 86.1 4 mo, 94.4 ± 61.1
Fields et al. [51]	2017	37	M	complete feed	8:00–10:00	1, 6 mo	SM	-	-	-	-	1 mo, 6.5 ± 0.8; 6 mo, 6.0 ± 1.0
Chan et al. [56]	2018	430	M	sample collected over 24 h and pooled	over 24 h, pooled together	4 mo	SM	ELISA		69		586 (340–1013), pg/mL
Young et al. [88]	2018	41	M	fasted, mid feed	morning	2 wk, 1, 2, 3, 4 mo	SM	RIA	-	-	-	2 wk, 93 ± 95.5; 1 mo, 185.4 ± 474; 2 mo, 88.2 ± 55.2; 3 mo, 87.6 ± 57.7; 4 mo, 81.6 ± 52.8
Yu et al. [57]	2018	96	C, M	pre- and post-feed for mature milk	3 d, 8:00–9:00 42, 90 d, 14:00–16:00	3, 42, 90 d	SM	ELISA	<4.1, 9.0	3		GDM (n = 48), d 3, 136.8 (81–307.5); d 42, 194.2 (78.4–349.3); d 90, 243.9 (134.9–343.1); Healthy (n = 48), d 3, 122.5 (46.1–188.3); d 42, 169.2 (107.4–240.3; d 90, 147.7 (80.4–191.1)
Sadr Dadres et al. [58]	2019	135	M	2 h after feed	10:00–12:00	1 and 3 mo	SM	ELISA	<6, 6	1.44	88–115	1 mo, 178.2 ± 134.4 (26.4–699) 3 mo, 19.2 ± 4.2 (9–28.8)
Grunewald et al. [90]	2019	367	M	-	-	16–163 d	SM	ELISA	3.4, 11	-	92.3 ± 14.8	86.5 ± 56.9
Schneider-Worthington et al. [64]	2020	25	M	-	-	1 mo	SM	ELISA	4.48, 13.56	-	-	60.06 (34.02)
Ellsworth et al. [115]	2020	32	M	2 h after feed	8:00–10:00	2 wk	SM	ELISA	-	-	-	NW, 100.6 ± 109.02 Ow/Ob, 204.86 ± 147.9
Mank et al. [116]	2021	32	M	-	-	1–10 mo	SM	CLIA	<6, -	-	101–117	(16–1431) (range)

Data reported as *mean ± SD or median (IQR)* unless specified. C, colostrum milk; CTL, control; CV %, coefficient variation percentage; CLIA, chemiluminescent immunoassay; CMIA, chemiluminescent microparticle immunoassay ECLIA, electrochemiluminescence immunoassay; ELISA, enzyme-linked immunosorbent assay; GDM, gestational diabetes mellitus; LOD, limit of detection; M, mature milk; mo, month; NW, normal weight; OW/Ob, with overweight/obesity; R%, recovery of analyte percentage; RIA, radioimmunoassay; wk, week.

**Table 5:** Studies examining human milk resistin.

Author	Year	Sample Size (n)	Sample Type	Sampling	Time of the Day (h)	Time of Collection	Preparation	Method	Intra, Inter Assay (%CV)	LOD (pg/mL)	R (%)	Concentration (pg/mL)
Ilcol et al. [120]	2008	160	C, M	3 h after breakfast	Morning	1–3, 4–14, 15–30, 30–90, 91–180 d	SM	ELISA	<5, 8	0.16	-	1710 ± 68
Savino et al. [37]	2012	23	-	-	-	<6 mo	SM	ELISA	<5, 6.8	6	-	180 (400)
Andreas et al. [46]	2016	120	C, M	1 h after feed, pre- and post-feed, protease inhibitors	10:00–13:00	1 wk, 3 mo	SM	Multiplex assay	3, 4	1.3	-	1 wk, pre-feed, 924.9 (295.9); post-feed, 967.4 (317.0) 3 mo, pre-feed, 792.5 (224.4); post-feed, 733.9 (238.9) mean (SEM); ng/mL

Data reported as mean ± SD or median (IQR) unless specified. C, colostrum milk; CV %, coefficient variation percentage; d, day; ELISA, enzyme-linked immunosorbent assay; LOD, limit of detection; M, mature milk; mo, month; R %, recovery of analyte percentage; SM, skim milk; wk, week.

**Table 6:** Studies examining human milk obestatin.

Author	Year	Sample Size (n)	Sample Type	Sampling	Time of the Day (h)	Time of Collection	Preparation	Method	Intra, Inter Assay (%CV)	LOD (pg/mL)	R (%)	Concentration (pg/mL)
Aydin et al. [31]	2008	31	C, M	before breakfast, on aprotinin	9:00	2, 25 d	SM	RIA	11.8, 13.6	76	92–124	C, 538.90 ± 46.21 M, 528.53 ± 39
Savino et al. [92]	2012	40	M	-	-	2–3 mo	SM	RIA	-	300–570	-	846.6 (472.07)

Data reported as mean ± SD or median (IQR). C, colostrum milk; M, mature milk; RIA, radioimmunoassay; d, day; mo, month; BMI: body mass index; CV %, coefficient variation percentage; LOD, limit of detection; R %, recovery of analyte percentage.

**Table 7:** Studies examining human milk apelin.

Author	Year	Sample Size (n)	Sample Type	Sampling	Time of the Day (h)	Time of Collection	Preparation	Method	Intra, Inter Assay (%CV)	LOD	R (%)	Concentration (ng/mL)
Aydin [102]	2010	20	C, M	-	08:00	1–4 mo	WM	EIA	-	-	-	GDM, C, apelin-12, 2.9 ± 0.6 C, apelin-36, 3.2 ± 0.7 M, apelin-12, 3.6 ± 1.2 M, apelin-36, 4.4 ± 1.4 CTL, C, apelin-12, 4.3 ± 1.2 C, apelin-36, 4.9 ± 2 M, apelin-12, 5.4 ± 1.8 M, apelin-36, 6.2 ± 1.9

Data reported as mean ± SD. C, colostrum milk; CTL, control group; CV %, coefficient variation percentage; EIA, enzyme immunoassay; GDM, gestational diabetes mellitus; LOD, limit of detection; M, mature milk; mo, month; R %, recovery of analyte percentage; WM, whole milk.

## Data Availability

Not applicable.

## Supplementary Materials

The Supplementary Tables S1–S7 are available online at https://www.mdpi.com/article/10.3390/ijms22168708/s1.
