# Supplementary material for: Human Milk Metabolic Hormones: Analytical Methods and Current Understanding
Source: Int J Mol Sci. 2021 Aug 13;22(16):8708. doi: 10.3390/ijms22168708 (PMC8395916; doi:10.3390/ijms22168708)
Supplement: Supplementary file 1 [file ijms-22-08708-s001.zip › ijms-1261897-supplementary.pdf]

Table S1. Summary of outcomes of studies examining human milk leptin.

Author	Samples size	Lactation stage	Outcomes/Effect
Houseknecht, et al. [1]	14	–	↑ Leptin concentration in whole milk vs skim milk<br>+ Correlation, HM leptin concentration and maternal plasma leptin, maternal body weight, BMI, and skinfold thickness
Uçar, et al. [2]	18	3-120 d	↑ Leptin in serum vs milk<br>+ Correlation, HM leptin, maternal and infant circulation leptin<br>No correlation, HM leptin, maternal, infant adiposity
Bielicki, et al. [3]	33	2-3, 4-5 d,<br>4-6 wk	↑ Leptin in term vs preterm milk<br>Moderate correlation, maternal and infant BMI, milk leptin<br>+ Correlation, maternal BMI, serum leptin<br>HM leptin decreases over the course of lactation
Dundar, et al. [4]	47	15 d,<br>1, 2, 3 mo	HM leptin, ↑ in LGA, ↓ in SGA vs AGA<br>+ Correlation, HM leptin, birth weight<br>– Correlation, WG in first 15 d and 1 mo, HM leptin<br>No correlation, HM leptin, maternal BMI
Ilcol, et al. [5]	160	2, 8, 25 d	HM leptin decreases over the course of lactation<br>+ Correlation, HM leptin, maternal BMI
Bronsky, et al. [6]	59	1-2 d	↑ Leptin in term vs preterm milk<br>+ Correlation, HM leptin, maternal pre-pregnancy weight and BMI, delivery weight and BMI
Miralles, et al. [7]	28	1, 3, 6, 9 mo	+ Correlation of HM leptin, maternal plasma leptin and BMI<br>– Correlation, HM leptin at 1 mo, infant BMI at 18 and 24 mo of age<br>– Correlation, HM leptin at 1 and 3 mo, infant BMI from 12 to 24 mo of age
Weyermann, et al. [8]	766	6 wk,<br>6 mo	+ Correlation, HM leptin and adiponectin<br>↑ HM leptin in mothers of females vs mothers of males
Weyermann, et al. [9]	767	6 wk	+ Correlation, HM leptin and adiponectin<br>+ Correlation, HM leptin, pre-pregnancy BMI.<br>↑ HM leptin in mothers of females vs mothers of males
Aydin, et al. [10]	31	2, 25 d	↑ Leptin in serum vs milk<br>+ Correlation, leptin in milk and serum
Savino, et al. [11]	36	< 6mo	HM leptin ↓ than infant and maternal serum leptin
Bronsky, et al. [12]	72	1 d,<br>1, 3, 6, 12 mo	↑ HM leptin on d 1 than at 1, 3, 6, and 12 mo<br>+ Correlation between adiponectin, AFABP, and leptin throughout the lactation
Schuster, et al. [13]	23	1, 2, 3, 4 wk	↑ Leptin in serum vs milk<br>+ Correlation, serum, milk leptin

Author	Samples size	Lactation stage	Outcomes/Effect
		2, 3, 4, 5, 6 mo	+ Correlation, serum and milk leptin, maternal BMI<br>– Correlation, milk leptin at 1wk, infant weight gain from end of 6wk
Eilers, et al. [14]	77	3 d, 28 d	No difference n leptin between preterm and term milk<br>Term HM leptin decreases from day 3 to 28<br>+ Correlation, HM leptin, maternal BMI
Fields and Demerath [15]	19	~ 1 mo	+ Correlation, maternal pre-pregnancy BMI, maternal BMI, HM leptin<br>↑ HM milk associated with lower infant BMI
Savino, et al. [16]	23	< 6 mo	+ Correlation, HM leptin and resistin
Schueler, et al. [17]	13	29-38 d	No difference leptin, pre- and post-feed<br>+ Correlation, HM leptin, maternal BMI and fat mass
Lönnerdal and Havel [18]	–	2-4 mo	Lipid in HM interfere with RIA
Chang, et al. [19]	–	3-4 mo	Leptin unaffected by the various handling procedures (fresh, frozen, pasteurized)
Ojeda, et al. [20]	–	2 mo	No difference in leptin concentration between ECI or ELISA
Kon, et al. [21]	103	1, 2, 3 mo	↑ HM leptin levels at 2 and 3 mo of lactation in infants with high weight gain (>1000 g/mo)
Brunner, et al. [22]	6 wk: 152<br>6 mo: 120	6 wk<br>6 mo	No relationship, HM leptin, infant anthropometrics up to 2 y
Khodabakhshi, et al. [23]	Ob: 40<br>NW: 40	2-5 mo	+ Correlation HM leptin, BMI of mother of infants with obesity<br>– Correlation HM leptin, weight of infants with normal weight at 2nd month
Cannon, et al. [24]	19	3-21 wk	Leptin significantly ↑ at night<br>Leptin is not associated with the time between feeds
Quinn, et al. [25]	113	10 d – 36 mo	+ Correlation, maternal % fat mass, HM leptin<br>HM leptin, predictor of infant WAZ, BMI z-score (< 1y)<br>– Correlation, HM leptin, both WAZ and BMI for age z-score in female but not male infants
Andreas, et al. [26]	120	1 wk,<br>3 mo	Pre-feed leptin concentration relates to maternal BMI at 1 wk and 3 mo<br>No difference in HM leptin pre- and post-feed
Gridneva, et al. [27]	27	2, 5 mo	No relationships, HM leptin concentration and dose with gastric emptying time or milk intake
Kugananthan, et al. [28]	61	2-12 mo	HM leptin ↑ in whole milk compared to skim milk
Resto, et al. [29]	29	1-4 wk	Birth gestational age, birth weight, and gender of the infant did not associate with HM leptin<br>↓ HM leptin with pasteurization
De Luca, et al. [30]	100	1 mo	↑ HM leptin concentration in mothers with obesity than in mothers with normal weight
Savino, et al. [31]	58	–	+ Correlation, circulation leptin, maternal BMI, HM leptin

Author	Samples size	Lactation stage	Outcomes/Effect
Fields, et al. [32]	37	1, 6 mo	+ Correlation, HM leptin, maternal BMI, infant length, infant body fat<br>↓ HM leptin from 1 to 6 mo
Meyer, et al. [33]	147	6 wk, 4 mo	HM leptin unrelated to child anthropometric measures at 3, 4, and 5y
Quinn and Childs [34]	116	–	+ Association, maternal BMI, HM leptin
Nunes, et al. [35]	69	1, 2, 30 d	↓ Leptin overtime, significant in mothers with SGA infants<br>+ Correlation, HM leptin, maternal BMI<br>No correlation, HM leptin, and infant WG at 1 month
Cannon, et al. [36]	20	–	No relationships, skim milk leptin concentration, dose with gastric emptying time and milk intake
Kugananthan, et al. [37]	59	2, 5, 9, 12 mo	+ Correlation, maternal % fat mass, HM leptin in whole and skim milk
Gridneva, et al. [38]	20	2, 5, 9, 12 mo	Intakes of HM leptin differentially influence development of infant BC in the first year of life
Chan, et al. [39]	430	4 mo	+ Correlation, HM leptin and maternal BMI<br>+ Correlation, HM leptin and infant WFL z-scores and BMI
Uysal, et al. [40]	50	1,2, 3 mo	+ Correlation of HM leptin, maternal BMI<br>No correlation, HM leptin, infant BMI
Yu, et al. [41]	96	3, 42, 90 d	HM leptin difference between GDM and healthy groups at 3rd d postpartum
Sadr Dadres, et al. [42]	135	1, 3 mo	↓ HM leptin from 1 to 3 mo<br>+ Correlation, pre-pregnancy BMI, HM leptin at 1 mo<br>+ Correlation, GWG, HM leptin<br>– Correlation, postpartum WG loss , HM leptin at 1 mo
Zamanillo, et al. [43]	59	30, 60, 90 d	Maternal obesity disturbs the breast milk supply of miRNAs<br>– Correlation between HM leptin and miRNAs in mothers with normal weight<br>No correlation in mothers with overweight/obesity
Logan, et al. [44]	SPATZ: 1090<br>UBCS: 1006	6 wk,<br>6 mo	In SPATZ, – correlation HM leptin, infant BMI at 6wk<br>In UBCS, not significant
Larrosa Haro, et al. [45]	131	8, 16 wk	↑ Leptin in pre-feed vs post-feed samples<br>↑ Leptin in serum than HM
Logan, et al. [64]	694	6 wk, 6 mo, 12 mo	Adiposity-related factors primarily determine HM leptin concentration; BMI, breastfeeding frequency, and HM fat concentration

Author	Samples size	Lactation stage	Outcomes/Effect
Kocaadam, et al. [47]	65	15-30 d	– Correlation, HM leptin, head circumference at birth in preterm infants, and the triceps skinfold thickness increment at 1 and 2 mo in term infants
Schneider-Worthington, et al. [48]	25	1 mo	+ Correlation, maternal circulation and HM leptin<br>+ Correlation, maternal fat mass, HM leptin
Galante, et al. [49]	501	2-3 mo	+ Correlation, HM leptin, maternal pre-pregnancy weight and BMI
Joung, et al. [50]	50	7, 14, 21, 28 d	↑ Leptin intake of preterm infants associate with ↑ WG, weight z-score, and height z-score at 36 weeks

AFABF, adipocyte fatty acid-binding protein; AGA, appropriate for gestational age; BC, body composition; BMI, body mass index; d, day; ECI, electrochemical immunosensor; EDTA, ethylenediaminetetraacetic acid; ELISA, enzyme-linked immunosorbent assay; GWG, gestational weight gain; HM, human milk; LGA, large for gestational age; mo, month; Non-Ob, without obesity; NW, with normal weight; Ob, with obesity; weight gain; RIA, radioimmunoassay; SGA, small for gestational age; SPATZ,ULM SPARTZ health study; UBCS, Ulm birth cohort study; WAZ, weight-for-age z score; WG, weight gain; wk, week; y, year; ↑, higher; ↓, lower; –. negative; +, positive.

Table S2. Summary of outcomes of studies examining human milk adiponectin.

Author	Sample size	Lactation stage	Outcomes/Effect
Bronsky, et al. [6]	59	1-2 d	+ Correlation, HM adiponectin, maternal body weight before delivery
Weyermann, et al. [8]	766	6 wk,<br>6 mo	No correlation, maternal serum, HM adiponectin<br>+ Correlation, HM leptin and adiponectin
Weyermann, et al. [9]	674	33-71 d	+ Correlation, HM leptin and adiponectin
Martin, et al. [51]	158	1 d - 12 mo	– Correlation, duration of lactation, HM adiponectin.<br>+ Correlation, maternal post-pregnancy BMI, HM adiponectin<br>HM adiponectin of Mexican mothers ↓ than in Hispanic mothers
Woo, et al. [52]	322	1wk - 6 mo	HM adiponectin associated with lower WAZ and WLZ but not LAZ
Dündar, et al. [53]	25	–	No relationship, HM adiponectin, BMI or birth weight of infants or BMI of mothers<br>No differences in adiponectin of colostrum, cord blood, maternal serum
Bronsky, et al. [12]	72	1 d,<br>1, 3, 6, 12 mo	↑ Adiponectin at 12 mo, compared with 3 and 6 mo<br>+ Correlation, adiponectin, AFABP, leptin
Luoto, et al. [54]	30	0 - 3 d	↑ Adiponectin in HM of mothers of children with normal weight vs overweight at 10 y of age<br>– Correlation, HM adiponectin, child BMI at 10 y<br>No correlation, HM adiponectin, pre-pregnancy BMI
Ley, et al. [55]	34	1-6 mo	No difference, whole vs skim milk adiponectin<br>Pasteurization reduces HM adiponectin by 32.8%
Cesur, et al. [56]	25	1, 4 mo	+ Correlation, HM adiponectin, infant serum adiponectin, infant weight gain

Author	Sample size	Lactation stage	Outcomes/Effect
			No difference HM adiponectin at 1 and 4 mo<br>No relationship, HM adiponectin, maternal and infant circulation adiponectin
Luoto, et al. [57]	256	0 - 3 d	Dietary intervention (diet and probiotics) ↑ HM adiponectin<br>– Correlation, HM adiponectin, maternal weight gain during pregnancy<br>No correlation, HM adiponectin, GDM
Ley, et al. [58]	170	2 d, 3 mo	↑ Adiponectin in colostrum than mature milk<br>No relationship, pre-pregnancy BMI, HM adiponectin
Liu, et al. [59]	48	3 d	↑ HM adiponectin in women with preeclampsia (PE) had than women without PE
Ozarda, et al. [60]	157	1 – 180 d	Skim milk most suitable for HM adiponectin analysis using RIA<br>HM adiponectin increase over lactation period<br>+ Correlation, HM adiponectin, infant serum adiponectin
Savino, et al. [61]	60	< 6 mo	+ Correlation, maternal serum adiponectin, HM adiponectin<br>– Correlation, infant age, HM adiponectin
Woo, et al. [62]	277	1wk,<br>1, 2, 5, 6 mo	+ Correlation, maternal serum adiponectin, HM adiponectin<br>↑ HM adiponectin - ↑ infant WAZ and WLZ between age 1 and 2 y
Kon, et al. [21]	103	1, 2, 3 mo	No difference in HM adiponectin concentration between groups (LWG, NWG, HWG), yet adiponectin CDI intake of adiponectin is higher in HWG than LWG group
Brunner, et al. [22]	6 wk: 152<br>6 mo: 120	6 wk,<br>6 mo	– Correlation, HM adiponectin and early infant anthropometrics up to 4 mo, then + correlation with infant weight gain and the sum of skinfolds up to 2 y of age<br>↑ Adiponectin at 4 wk than 6 mo
Khodabakhshi, et al. [23]	Ob: 40<br>NW: 40	2-5 mo	No difference in HM adiponectin between mothers of infants with obesity and normal weight<br>↑ HM adiponectin – lower infant weight at 4th month in infants with obesity only (-0.354, $p<0.05$; provided by author on request)
Gridneva, et al. [27]	27	2 and 5 mo	↑ HM adiponectin concentration and dose – longer gastric emptying time in term infants
Anderson, et al. [63]	117	9 d - 24 mo	No difference, maternal body composition, HM adiponectin<br>+ Correlation, HM adiponectin, infant WAZ and WLZ
Nunes, et al. [35]	69	1, 2, 30 d	No relationships, maternal BMI, HM adiponectin
Quinn and Childs [34]	116	–	No relationships, HM adiponectin, maternal BMI<br>– Correlation, HM adiponectin, infant WAZ<br>↓ HM adiponectin in the Tibetans (lower altitude) than in Nepal (higher altitude) population
Kugananthan, et al. [37]	59	2, 5, 9, 12 mo	No relationships, HM adiponectin, maternal % fat mass<br>No significant difference in HM adiponectin over the first year of lactation
Gridneva, et al. [38]	20	2, 5, 9, 12 mo	↑ Calculated daily intakes of HM adiponectin – lower infant fat-free mass and fat-free-mass index, higher infant fat mass, % fat mass and fat mass index
Chan, et al. [39]	430	4 mo	No relationship, HM adiponectin, maternal BMI, infant body composition

Author	Sample size	Lactation stage	Outcomes/Effect
Yu, et al. [41]	96	3, 42, 90 d	– Correlation, GDM, HM adiponectin<br>– Correlation, HM adiponectin, infants WLZ, head circumference in both groups (with and without GDM)
Young, et al. [64]	41	2 wk,<br>1,2, 3, 4 mo	+ Correlation, maternal circulation adiponectin, HM adiponectin at 2wk, 4 mo<br>HM adiponectin decreases over the lactation period<br>No difference in HM adiponectin, between groups with normal weight and overweight/obesity
Mohamad, et al. [65]	155	1d, 2mo	– Correlation, HM adiponectin, infant weight, BMI-for-age Z scores, abdominal circumference
Sadr Dadres, et al. [42]	135	1, 3 mo	– Correlation, pre-pregnancy BMI, HM adiponectin, "the relationship decreased over time, close to zero at 3 months"
Zamanillo, et al. [43]	59	30, 60, 90 d	Adiponectin decreases over lactation period in mothers with normal weight, but not in mothers with obesity<br>– Correlation, miRNAs and HM adiponectin in mothers with normal weight, but not in mothers with obesity
Grunewald, et al. [66]	367	16, 163 d	+ Correlation, adiponectin with HM protein<br>Adiponectin decreases over lactation period
Kocaadam, et al. [47]	65	15-30 d	– Correlation, HM adiponectin, term infant anthropometrics<br>+ Correlation, HM adiponectin, preterm BMI in 2 and 3 mo<br>– Correlation, HM adiponectin and preterm infant length
Schneider-Worthington, et al. [48]	25	1 mo	+ Correlation, maternal circulation and HM adiponectin
Galante, et al. [49]	501	2-3 mo	No correlation, maternal factors, HM adiponectin<br>+ Correlation, HM adiponectin, infant birth weight and giving birth to twins
Joung, et al. [50]	50	7, 14, 21, 28 d	+ Correlation of HM adiponectin, length z-score at 36 weeks; correlation confounded by total protein and calorie intake<br>+ Correlation HM adiponectin with head circumference

AFABF, adipocyte fatty acid-binding protein; BMI, body mass index; GDM, gestational diabetes; HM, human milk; HWG, high weight gain; LAZ, Length-for-age z score; LWG, low weight gain; mo, month; NW, normal weight; NWG, normal weight gain; Ob, with obesity; RIA, radioimmunoassay; WAZ, weight-for-age z score; WG, weight gain.; wk, Week; WLZ, weight-for-length z score; ↑, higher; ↓, lower; –, negative; +, positive.

Table S3. Summary of outcomes of studies examining human milk ghrelin.

Author	Sample size	Lactation stage	Outcomes/Effect
Aydin, et al. [67]	17	1, 7, 15 d	HM Gh concentration increases over lactation<br>HM Gh increases as maternal plasma ghrelin increases after delivery
Kierson, et al. [68]	10	7-21 d	↑ HM Gh than in maternal serum<br>+ Correlation, whole HM Gh, HM fat<br>↑ Gh in whole than skim milk
Aydin, et al. [69]	29	2, 15 d	dGh 24-fold ↑ than aGh using HPLC<br>Mothers with GDM have 2-fold ↓ HM and serum aGh than mothers without GDM at 2 d postpartum
Ilcol and Hizli [70]	159	1-3, 4-14, 15-30<br>30-90, 91-180 d	aGh and tGh increase over lactation period
Aydin, et al. [10]	31	2, 25 d	– Correlation HM Gh, maternal BMI<br>+ Correlation, HM Gh, maternal serum Gh
Aydin [71]	20	NA	dGh forms most of the HM Gh<br>– Correlation, maternal BMI, HM Gh
Dündar, et al. [53]	25	–	No differences, tGh levels in colostrum, cord blood, maternal serum<br>– Correlation, tGh and infant BMI, birth weight<br>+ Correlation, aGh, tGh and maternal serum Gh
Yis, et al. [72]	47	3-4 mo	↑ HM Gh - ↑ infant growth rate during the first 3 mo of age
Karatas, et al. [73]	46	1-3 mo,<br>4-6 mo	↓ tGh and aGh in the pre- than post-feed sample<br>Pre-feed sample: ↓ tGh and ↑ aGh at 4-6 mo compared with 1-3 mo
Savino, et al. [74]	20	1-5 mo	↑ Gh in formula vs HM<br>+ Correlation, milk Gh, infant serum Gh<br>↑ Gh in serum of formula fed infants than breastfed infants
Cesur, et al. [56]	25	1, 4 mo	↑ HM and infant serum aGh at 4mo vs 1mo<br>↑ HM aGh vs infant and maternal serum
Savino, et al. [75]	40	2-3 mo	+ Correlation, maternal serum Gh and HM Gh, maternal and breastfed infants serum Gh, HM Gh and breastfed infants serum Gh
Kon, et al. [21]	103	1, 2, 3 mo	+ Correlation, HM Gh and infant serum Gh<br>↑ Gh at 1 and 2 mo in serum of infants with high weight gain
Khodabakhshi, et al. [23]	Ob: 40<br>NW: 40	2-5 mo	HM Gh ↑ in mothers of infant with normal weight vs mothers of infants with obesity
Andreas, et al. [26]	120	1 wk,<br>3 mo	HM Gh decreases over feed<br>HM Gh decreases over lactation period, significant for post-feed<br>HM Gh not related to maternal BMI

Author	Sample size	Lactation stage	Outcomes/Effect
Slupecka-Ziemilska, et al. [76]	40	3 d	↓ HM Gh in milk vs maternal plasma<br>Mammary gland is a source of HM Gh
Young, et al. [77]	48	2 wk,<br>1, 2, 3, 4 mo	HM Gh decreases over lactation<br>No difference of HM Gh between mothers of infant with normal weight and mothers of infants with overweight/obesity
Yu, et al. [41]	96	3, 42, 90 d	↓ HM Gh in mothers with GDM<br>– Correlation, maternal BMI, HM Gh<br>No correlation, HM Gh, infant head circumference and WHZ
Larrosa Haro, et al. [45]	131	8, 16 wk	↑ HM Gh in post-feed sample vs pre-feed sample<br>↑ Gh in maternal serum than in pre-feed sample

aGh, acylated-ghrelin; BMI, body mass index; dGh, deacylated-ghrelin; GDM, gestational diabetes mellitus; Gh, ghrelin; HM, human milk; HPLC, high pressure liquid chromatography; mo, month; NW, with normal weight; Ob, with obesity; tGh, total ghrelin; WHZ, weight-for-height z-score; wk, week; ↑, higher; ↓, lower; –, negative; +, positive.

Table S4. Summary of outcomes of studies examining human milk insulin.

Author	Sample size	Lactation stage	Outcomes/Effect
Shehadeh, et al. [78]	90	3, 10 d	HM insulin not related to gestational age, preterm birth
Ley, et al. [55]	34	1-6 mo	Pasteurization reduces HM insulin concentration by 46.1%
Ley, et al. [58]	170	2 d, 3 mo	High HM leptin in colostrum vs mature milk<br>↑ Pre-pregnancy BMI, gestational weight gain associated with ↑ insulin in mature milk, but not in colostrum<br>No correlation between GDM and HM insulin (in colostrum and mature milk)
Fields and Demerath [15]	19	1 mo	↑ HM insulin associated with lower infant weight, relative weight, and lean mass
Whitmore, et al. [79]	14	1-6 mo	No variation in HM insulin concentration over 24-hours<br>No significant difference in HM insulin concentration between pre- and post-sample
Andreas, et al. [26]	120	1 wk, 3 mo	+ Correlation, maternal BMI and pre-feed insulin at 3 mo<br>HM insulin concentration decreases from pre to post-feed
Nunes, et al. [35]	69	1, 2, 30 d	– Correlation between HM insulin and infant weight gain at 1 mo<br>+ Correlation between mature HM insulin and maternal BMI<br>Insulin concentration decreases over lactation period
Young, et al. [77]	48	2 wk,<br>1, 2, 3, 4 mo	HM insulin is ↑ in mothers with overweight/obesity compared to mothers with normal weight<br>HM insulin ↑ than maternal plasma insulin<br>+ Correlation between maternal fasting plasma insulin and HM insulin
Fields, et al. [32]	37	1, 6 mo	HM insulin ↑ in mothers with obesity than in mothers with normal weight

Author	Sample size	Lactation stage	Outcomes/Effect
			229% higher HM insulin in mothers with obesity nursing female infants vs mothers with normal weight nursing female infants, and 179% higher than in mothers with obesity nursing male infants
Chan, et al. [39]	430	4 mo	+ Correlation, pre-pregnancy BMI and HM insulin<br>Ethnicity, Asian mothers have lower HM insulin compared to Caucasian mothers
Young, et al. [64]	41	2 wk,<br>1, 2, 3, 4 mo	– Correlation between HM insulin and WLZ trajectory among infants of mothers with normal weight
Yu, et al. [41]	96	3, 42, 90 d	↑ HM insulin in mothers with GDM<br>+ Correlation, maternal BMI and HM insulin
Sadr Dadres, et al. [42]	135	1, 3 mo	+ Correlation, pre-pregnancy BMI and HM insulin at 1st and 3rd mo
Grunewald, et al. [66]	367	16-163 d	+ Correlation, pre-pregnancy BMI and HM insulin
Schneider-Worthington, et al. [48]	25	1 mo	+ Correlation, maternal circulation and HM insulin<br>+ Correlation, maternal fat mass and HM insulin
Ellsworth, et al. [80]	32	2 wk	↑ HM insulin concentrations n mothers with overweight and obesity<br>+ Correlation, HM insulin and infant WFA from 2 wk to 6 mo and HCA z-score change from 2 wk to 2 mo

BMI, body mass index; GDM, gestational diabetes mellitus; HCA, head circumference-for-age; HM, human milk; mo, month; WFA: weight-for-age; wk, Week; WLZ, weight for length z-score; ↑, higher; ↓, lower; –, negative; +, positive.

Table S5. Summary of outcomes of studies examining human milk resistin.

Author	Sample size	Lactation stage	Outcomes/Effect
Ilcol, et al. [81]	160	1-3, 4-14, 15-30<br>30-90, 91-180 d	HM and maternal serum resistin concentrations decrease gradually from 1 to 3 d until 180 d postpartum<br>+ Correlation, maternal serum and HM resistin
Savino, et al. [16]	23	< 6 mo	+ Correlation, HM resistin and breastfed infants' serum resistin
Andreas, et al. [26]	120	1 wk, 3 mo	Resistin concentration shows no difference over a feed but decreases over lactation period

HM, human milk; mo, month; wk, week; d, day; +, positive.

Table S6. Summary of outcomes of studies examining human milk obestatin.

Ref.	Sample size	Lactation stage	Outcomes/Effect
Aydin, et al. [10]	31	2, 25 day	↑ HM obestatin than maternal circulation obestatin<br>No correlation between maternal BMI and HM obestatin
Savino, et al. [75]	40	2-3 mo	+ Correlation, HM obestatin and infants' age, HM obestatin and maternal serum obestatin

HM, human milk; mo, month; BMI, body mass index; ↑, higher; +, positive.

Table S7. Summary of outcomes of studies examining human milk apelin.

Author	Sample size	Lactation stage	Outcomes/effect
Aydin [71]	20	1 – 4 mo	↓ HM apelin in women with GDM than in mothers without GDM<br>+ Correlations, colostrum and mature milk apelin concentrations, and mature milk and maternal serum

HM, human milk; mo, month; GDM, gestational diabetes mellitus; ↓, lower, +, positive.

References

1. Houseknecht, K.L.; McGuire, M.K.; Portocarrero, C.P.; McGuire, M.A.; Beerman, K. Leptin is present in human milk and is related to maternal plasma leptin concentration and adiposity. *Biochemical and biophysical research communications* 1997, *240*, 742-747, doi:10.1006/bbrc.1997.7736.
2. Uçar, B.; Kirel, B.; Bör, O.; Kiliç, F.S.; Doğruel, N.; Aydoğdu, S.D.; Tekin, N. Breast milk leptin concentrations in initial and terminal milk samples: relationships to maternal and infant plasma leptin concentrations, adiposity, serum glucose, insulin, lipid and lipoprotein levels. *Journal of pediatric endocrinology & metabolism : JPEM* 2000, *13*, 149-156, doi:10.1515/jpem.2000.13.2.149.
3. Bielicki, J.; Huch, R.; von Mandach, U. Time-course of leptin levels in term and preterm human milk. *European journal of endocrinology* 2004, *151*, 271-276, doi:10.1530/eje.0.1510271.
4. Dundar, N.O.; Anal, O.; Dundar, B.; Ozkan, H.; Caliskan, S.; Büyükgebiz, A. Longitudinal investigation of the relationship between breast milk leptin levels and growth in breast-fed infants. *Journal of pediatric endocrinology & metabolism : JPEM* 2005, *18*, 181-187, doi:10.1515/jpem.2005.18.2.181.
5. Ilcol, Y.O.; Hizli, Z.B.; Ozkan, T. Leptin concentration in breast milk and its relationship to duration of lactation and hormonal status. *International breastfeeding journal* 2006, *1*, 21, doi:10.1186/1746-4358-1-21.
6. Bronsky, J.; Karpísek, M.; Bronská, E.; Pechová, M.; Jancíková, B.; Kotolová, H.; Stejskal, D.; Prusa, R.; Nevoral, J. Adiponectin, adipocyte fatty acid binding protein, and epidermal fatty acid binding protein: proteins newly identified in human breast milk. *Clinical chemistry* 2006, *52*, 1763-1770, doi:10.1373/clinchem.2005.063032.
7. Miralles, O.; Sánchez, J.; Palou, A.; Picó, C. A physiological role of breast milk leptin in body weight control in developing infants. *Obesity (Silver Spring, Md.)* 2006, *14*, 1371-1377, doi:10.1038/oby.2006.155.
8. Weyermann, M.; Beermann, C.; Brenner, H.; Rothenbacher, D. Adiponectin and leptin in maternal serum, cord blood, and breast milk. *Clinical chemistry* 2006, *52*, 2095-2102, doi:10.1373/clinchem.2006.071019.
9. Weyermann, M.; Brenner, H.; Rothenbacher, D. Adipokines in human milk and risk of overweight in early childhood: a prospective cohort study. *Epidemiology (Cambridge, Mass.)* 2007, *18*, 722-729, doi:10.1097/ede.0b013e3181567ed4.
10. Aydin, S.; Ozkan, Y.; Erman, F.; Gurates, B.; Kilic, N.; Colak, R.; Gundogan, T.; Catak, Z.; Bozkurt, M.; Akin, O.; et al. Presence of obestatin in breast milk: relationship among obestatin, ghrelin, and leptin in lactating women. *Nutrition (Burbank, Los Angeles County, Calif.)* 2008, *24*, 689-693, doi:10.1016/j.nut.2008.03.020.

11. Savino, F.; Liguori, S.A.; Petrucci, E.; Lupica, M.M.; Fissore, M.F.; Oggero, R.; Silvestro, L. Evaluation of leptin in breast milk, lactating mothers and their infants. *European journal of clinical nutrition* 2010, *64*, 972-977, doi:10.1038/ejcn.2010.105.
12. Bronsky, J.; Mitrova, K.; Karpisek, M.; Mazoch, J.; Durilova, M.; Fisarkova, B.; Stechova, K.; Prusa, R.; Nevoral, J. Adiponectin, AFABP, and leptin in human breast milk during 12 months of lactation. *Journal of pediatric gastroenterology and nutrition* 2011, *52*, 474-477, doi:10.1097/MPG.0b013e3182062fcc.
13. Schuster, S.; Hechler, C.; Gebauer, C.; Kiess, W.; Kratzsch, J. Leptin in maternal serum and breast milk: association with infants' body weight gain in a longitudinal study over 6 months of lactation. *Pediatric research* 2011, *70*, 633-637, doi:10.1203/PDR.0b013e31823214ea.
14. Eilers, E.; Ziska, T.; Harder, T.; Plagemann, A.; Obladen, M.; Loui, A. Leptin determination in colostrum and early human milk from mothers of preterm and term infants. *Early human development* 2011, *87*, 415-419, doi:10.1016/j.earlhumdev.2011.03.004.
15. Fields, D.A.; Demerath, E.W. Relationship of insulin, glucose, leptin, IL-6 and TNF-alpha in human breast milk with infant growth and body composition. *Pediatr Obes* 2012, *7*, 304-312, doi:10.1111/j.2047-6310.2012.00059.x.
16. Savino, F.; Sorrenti, M.; Benetti, S.; Lupica, M.M.; Liguori, S.A.; Oggero, R. Resistin and leptin in breast milk and infants in early life. *Early human development* 2012, *88*, 779-782, doi:10.1016/j.earlhumdev.2012.05.004.
17. Schueler, J.; Alexander, B.; Hart, A.M.; Austin, K.; Larson-Meyer, D.E. Presence and dynamics of leptin, GLP-1, and PYY in human breast milk at early postpartum. *Obesity (Silver Spring, Md.)* 2013, *21*, 1451-1458, doi:10.1002/oby.20345.
18. Lönnerdal, B.; Havel, P.J. Serum leptin concentrations in infants: effects of diet, sex, and adiposity. *The American journal of clinical nutrition* 2000, *72*, 484-489, doi:10.1093/ajcn/72.2.484.
19. Chang, J.C.; Chen, C.H.; Fang, L.J.; Tsai, C.R.; Chang, Y.C.; Wang, T.M. Influence of prolonged storage process, pasteurization, and heat treatment on biologically-active human milk proteins. *Pediatrics and neonatology* 2013, *54*, 360-366, doi:10.1016/j.pedneo.2013.03.018.
20. Ojeda, I.; Moreno-Guzmán, M.; González-Cortés, A.; Yáñez-Sedeño, P.; Pingarrón, J.M. A disposable electrochemical immunosensor for the determination of leptin in serum and breast milk. *The Analyst* 2013, *138*, 4284-4291, doi:10.1039/c3an00183k.
21. Kon, I.Y.; Shilina, N.M.; Gmoshinskaya, M.V.; Ivanushkina, T.A. The study of breast milk IGF-1, leptin, ghrelin and adiponectin levels as possible reasons of high weight gain in breast-fed infants. *Annals of nutrition & metabolism* 2014, *65*, 317-323, doi:10.1159/000367998.
22. Brunner, S.; Schmid, D.; Zang, K.; Much, D.; Knoeferl, B.; Kratzsch, J.; Amann-Gassner, U.; Bader, B.L.; Hauner, H. Breast milk leptin and adiponectin in relation to infant body composition up to 2 years. *Pediatric obesity* 2014, *10*, 67-73, doi:10.1111/j.2047-6310.2014.222.x.
23. Khodabakhshi, A.; Ghayour-Mobarhan, M.; Rooki, H.; Vakili, R.; Hashemy, S.I.; Mirhafez, S.R.; Shakeri, M.T.; Kashanifar, R.; Pourbafarani, R.; Mirzaei, H.; et al. Comparative measurement of ghrelin, leptin, adiponectin, EGF and IGF-1 in breast milk of mothers with overweight/obese and normal-weight infants. *European journal of clinical nutrition* 2015, *69*, 614-618, doi:10.1038/ejcn.2014.205.
24. Cannon, A.M.; Kakulas, F.; Hepworth, A.R.; Lai, C.T.; Hartmann, P.E.; Geddes, D.T. The Effects of Leptin on Breastfeeding Behaviour. *International journal of environmental research and public health* 2015, *12*, 12340-12355, doi:10.3390/ijerph121012340.
25. Quinn, E.A.; Largado, F.; Borja, J.B.; Kuzawa, C.W. Maternal characteristics associated with milk leptin content in a sample of Filipino women and associations with infant weight for age. *Journal of human lactation : official journal of International Lactation Consultant Association* 2015, *31*, 273-281, doi:10.1177/0890334414553247.
26. Andreas, N.J.; Hyde, M.J.; Herbert, B.R.; Jeffries, S.; Santhakumaran, S.; Mandalia, S.; Holmes, E.; Modi, N. Impact of maternal BMI and sampling strategy on the concentration of leptin, insulin, ghrelin and resistin in breast milk across a single feed: a longitudinal cohort study. *BMJ open* 2016, *6*, e010778, doi:10.1136/bmjopen-2015-010778.
27. Gridneva, Z.; Kugananthan, S.; Hepworth, A.R.; Tie, W.J.; Lai, C.T.; Ward, L.C.; Hartmann, P.E.; Geddes, D.T. Effect of Human Milk Appetite Hormones, Macronutrients, and Infant Characteristics on Gastric Emptying and Breastfeeding Patterns of Term Fully Breastfed Infants. *Nutrients* 2016, *9*, doi:10.3390/nu9010015.
28. Kugananthan, S.; Lai, C.T.; Gridneva, Z.; Mark, P.J.; Geddes, D.T.; Kakulas, F. Leptin Levels Are Higher in Whole Compared to Skim Human Milk, Supporting a Cellular Contribution. *Nutrients* 2016, *8*, doi:10.3390/nu8110711.
29. Resto, M.; O'Connor, D.; Leef, K.; Funanage, V.; Spear, M.; Locke, R. Leptin levels in preterm human breast milk and infant formula. *Pediatrics* 2001, *108*, E15, doi:10.1542/peds.108.1.e15.
30. De Luca, A.; Frasquet-Darrieux, M.; Gaud, M.A.; Christin, P.; Boquien, C.Y.; Millet, C.; Herviou, M.; Darmaun, D.; Robins, R.J.; Ingrand, P.; et al. Higher Leptin but Not Human Milk Macronutrient Concentration Distinguishes Normal-Weight from Obese Mothers at 1-Month Postpartum. *PloS one* 2016, *11*, e0168568, doi:10.1371/journal.pone.0168568.
31. Savino, F.; Sardo, A.; Rossi, L.; Benetti, S.; Savino, A.; Silvestro, L. Mother and Infant Body Mass Index, Breast Milk Leptin and Their Serum Leptin Values. *Nutrients* 2016, *8*, doi:10.3390/nu8060383.
32. Fields, D.A.; George, B.; Williams, M.; Whitaker, K.; Allison, D.B.; Teague, A.; Demerath, E.W. Associations between human breast milk hormones and adipocytokines and infant growth and body composition in the first 6 months of life. *Pediatric obesity* 2017, *12 Suppl 1*, 78-85, doi:10.1111/ijpo.12182.

33. Meyer, D.M.; Brei, C.; Stecher, L.; Much, D.; Brunner, S.; Hauner, H. The relationship between breast milk leptin and adiponectin with child body composition from 3 to 5 years: a follow-up study. *Pediatric obesity* 2017, *12 Suppl 1*, 125-129, doi:10.1111/ijpo.12192.
34. Quinn, E.A.; Childs, G. Ecological pressures and milk metabolic hormones of ethnic Tibetans living at different altitudes. *Annals of human biology* 2017, *44*, 34-45, doi:10.3109/03014460.2016.1153144.
35. Nunes, M.; da Silva, C.H.; Bosa, V.L.; Bernardi, J.R.; Werlang, I.C.R.; Goldani, M.Z.; Group, N. Could a remarkable decrease in leptin and insulin levels from colostrum to mature milk contribute to early growth catch-up of SGA infants? *BMC Pregnancy Childbirth* 2017, *17*, 410, doi:10.1186/s12884-017-1593-0.
36. Cannon, A.M.; Gridneva, Z.; Hepworth, A.R.; Lai, C.T.; Tie, W.J.; Khan, S.; Hartmann, P.E.; Geddes, D.T. The relationship of human milk leptin and macronutrients with gastric emptying in term breastfed infants. *Pediatric research* 2017, *82*, 72-78, doi:10.1038/pr.2017.79.
37. Kugananthan, S.; Gridneva, Z.; Lai, C.T.; Hepworth, A.R.; Mark, P.J.; Kakulas, F.; Geddes, D.T. Associations between Maternal Body Composition and Appetite Hormones and Macronutrients in Human Milk. *Nutrients* 2017, *9*, doi:10.3390/nu9030252.
38. Gridneva, Z.; Kugananthan, S.; Rea, A.; Lai, C.T.; Ward, L.C.; Murray, K.; Hartmann, P.E.; Geddes, D.T. Human Milk Adiponectin and Leptin and Infant Body Composition over the First 12 Months of Lactation. *Nutrients* 2018, *10*, doi:10.3390/nu10081125.
39. Chan, D.; Goruk, S.; Becker, A.B.; Subbarao, P.; Mandhane, P.J.; Turvey, S.E.; Lefebvre, D.; Sears, M.R.; Field, C.J.; Azad, M.B. Adiponectin, leptin and insulin in breast milk: associations with maternal characteristics and infant body composition in the first year of life. *International journal of obesity (2005)* 2018, *42*, 36-43, doi:10.1038/ijo.2017.189.
40. Uysal, F.K.; Onal, E.E.; Aral, Y.Z.; Adam, B.; Dilmen, U.; Ardiçolu, Y. Breast milk leptin: its relationship to maternal and infant adiposity. *Clinical nutrition (Edinburgh, Scotland)* 2002, *21*, 157-160, doi:10.1054/clnu.2001.0525.
41. Yu, X.; Rong, S.S.; Sun, X.; Ding, G.; Wan, W.; Zou, L.; Wu, S.; Li, M.; Wang, D. Associations of breast milk adiponectin, leptin, insulin and ghrelin with maternal characteristics and early infant growth: a longitudinal study. *Br J Nutr* 2018, *120*, 1380-1387, doi:10.1017/s0007114518002933.
42. Sadr Dadres, G.; Whitaker, K.M.; Haapala, J.L.; Foster, L.; Smith, K.D.; Teague, A.M.; Jacobs, D.R., Jr.; Kharbanda, E.O.; McGovern, P.M.; Schoenfuss, T.C.; et al. Relationship of Maternal Weight Status Before, During, and After Pregnancy with Breast Milk Hormone Concentrations. *Obesity (Silver Spring, Md.)* 2019, *27*, 621-628, doi:10.1002/oby.22409.
43. Zamanillo, R.; Sánchez, J.; Serra, F.; Palou, A. Breast Milk Supply of MicroRNA Associated with Leptin and Adiponectin Is Affected by Maternal Overweight/Obesity and Influences Infancy BMI. *Nutrients* 2019, *11*, doi:10.3390/nu11112589.
44. Logan, C.A.; Siziba, L.P.; Koenig, W.; Carr, P.; Brenner, H.; Rothenbacher, D.; Genuneit, J. Leptin in Human Milk and Child Body Mass Index: Results of the Ulm Birth Cohort Studies. *Nutrients* 2019, *11*, doi:10.3390/nu11081883.
45. Larrosa Haro, A.; Vásquez-Garibay, E.M.; Guzmán-Mercado, E.; Muñoz Esparza, N.C.; García-Arellano, S.; Muñoz-Valle, J.F.; Romero-Velarde, E. Concentration of ghrelin and leptin in serum and human milk in nursing mothers according to the type of feeding. *Nutricion hospitalaria* 2019, *36*, 799-804, doi:10.20960/nh.02534.
46. Logan, C.A.; Koenig, W.; Rothenbacher, D.; Genuneit, J. Determinants of leptin in human breast milk: results of the Ulm SPATZ Health Study. *International journal of obesity (2005)* 2019, *43*, 1174-1180, doi:10.1038/s41366-018-0200-4.
47. Kocaadam, B.; Koksal, E.; Ozcan, K.E.; Turkyilmaz, C. Do the adiponectin and leptin levels in preterm and term breast milk samples relate to infants' short-term growth? *Journal of developmental origins of health and disease* 2019, *10*, 253-258, doi:10.1017/s2040174418000703.
48. Schneider-Worthington, C.R.; Bahorski, J.S.; Fields, D.A.; Gower, B.A.; Fernandez, J.R.; Chandler-Laney, P.C. Associations Among Maternal Adiposity, Insulin, and Adipokines in Circulation and Human Milk. *Journal of human lactation : official journal of International Lactation Consultant Association* 2020, 890334420962711, doi:10.1177/0890334420962711.
49. Galante, L.; Lagström, H.; Vickers, M.H.; Reynolds, C.M.; Rautava, S.; Milan, A.M.; Cameron-Smith, D.; Pundir, S. Sexually Dimorphic Associations between Maternal Factors and Human Milk Hormonal Concentrations. *Nutrients* 2020, *12*, 152, doi:10.3390/nu12010152.
50. Joung, K.E.; Martin, C.R.; Cherkerzian, S.; Kellogg, M.; Belfort, M.B. Human milk hormone intake in the first month of life and physical growth outcomes in preterm infants. *The Journal of clinical endocrinology and metabolism* 2021, doi:10.1210/clinem/dgab001.
51. Martin, L.J.; Woo, J.G.; Geraghty, S.R.; Altaye, M.; Davidson, B.S.; Banach, W.; Dolan, L.M.; Ruiz-Palacios, G.M.; Morrow, A.L. Adiponectin is present in human milk and is associated with maternal factors. *The American journal of clinical nutrition* 2006, *83*, 1106-1111, doi:10.1093/ajcn/83.5.1106.
52. Woo, J.G.; Guerrero, M.L.; Altaye, M.; Ruiz-Palacios, G.M.; Martin, L.J.; Dubert-Ferrandon, A.; Newburg, D.S.; Morrow, A.L. Human milk adiponectin is associated with infant growth in two independent cohorts. *Breastfeeding medicine : the official journal of the Academy of Breastfeeding Medicine* 2009, *4*, 101-109, doi:10.1089/bfm.2008.0137.
53. Dündar, N.O.; Dündar, B.; Cesur, G.; Yilmaz, N.; Sütçu, R.; Ozgüner, F. Ghrelin and adiponectin levels in colostrum, cord blood and maternal serum. *Pediatrics international : official journal of the Japan Pediatric Society* 2010, *52*, 622-625, doi:10.1111/j.1442-200X.2010.03100.x.
54. Luoto, R.; Kalliomaki, M.; Laitinen, K.; Delzenne, N.M.; Cani, P.D.; Salminen, S.; Isolauri, E. Initial dietary and microbiological environments deviate in normal-weight compared to overweight children at 10 years of age. *Journal of pediatric gastroenterology and nutrition* 2011, *52*, 90-95, doi:10.1097/MPG.0b013e3181f3457f.

55. Ley, S.H.; Hanley, A.J.; Stone, D.; O'Connor, D.L. Effects of pasteurization on adiponectin and insulin concentrations in donor human milk. *Pediatric research* 2011, *70*, 278-281, doi:10.1203/PDR.0b013e318224287a.
56. Cesur, G.; Ozguner, F.; Yilmaz, N.; Dundar, B. The relationship between ghrelin and adiponectin levels in breast milk and infant serum and growth of infants during early postnatal life. *The journal of physiological sciences : JPS* 2012, *62*, 185-190, doi:10.1007/s12576-012-0193-z.
57. Luoto, R.; Laitinen, K.; Nermes, M.; Isolauri, E. Impact of maternal probiotic-supplemented dietary counseling during pregnancy on colostrum adiponectin concentration: a prospective, randomized, placebo-controlled study. *Early Hum Dev* 2012, *88*, 339-344, doi:10.1016/j.earlhumdev.2011.09.006.
58. Ley, S.H.; Hanley, A.J.; Sermer, M.; Zinman, B.; O'Connor, D.L. Associations of prenatal metabolic abnormalities with insulin and adiponectin concentrations in human milk. *The American journal of clinical nutrition* 2012, *95*, 867-874, doi:10.3945/ajcn.111.028431.
59. Liu, Y.; Zhu, L.; Pan, Y.; Sun, L.; Chen, D.; Li, X. Adiponectin levels in circulation and breast milk and mRNA expression in adipose tissue of preeclampsia women. *Hypertension in pregnancy* 2012, *31*, 40-49, doi:10.3109/10641955.2010.525273.
60. Ozarda, Y.; Gunes, Y.; Tuncer, G.O. The concentration of adiponectin in breast milk is related to maternal hormonal and inflammatory status during 6 months of lactation. *Clinical chemistry and laboratory medicine* 2012, *50*, 911-917, doi:10.1515/cclm-2011-0724.
61. Savino, F.; Lupica, M.M.; Benetti, S.; Petrucci, E.; Liguori, S.A.; Cordero Di Montezemolo, L. Adiponectin in breast milk: relation to serum adiponectin concentration in lactating mothers and their infants. *Acta paediatrica (Oslo, Norway : 1992)* 2012, *101*, 1058-1062, doi:10.1111/j.1651-2227.2012.02744.x.
62. Woo, J.G.; Guerrero, M.L.; Guo, F.; Martin, L.J.; Davidson, B.S.; Ortega, H.; Ruiz-Palacios, G.M.; Morrow, A.L. Human milk adiponectin affects infant weight trajectory during the second year of life. *Journal of pediatric gastroenterology and nutrition* 2012, *54*, 532-539, doi:10.1097/MPG.0b013e31823fde04.
63. Anderson, J.; McKinley, K.; Onugha, J.; Duazo, P.; Chernoff, M.; Quinn, E.A. Lower levels of human milk adiponectin predict offspring weight for age: a study in a lean population of Filipinos. *Maternal & child nutrition* 2016, *12*, 790-800, doi:10.1111/mcn.12216.
64. Young, B.E.; Levek, C.; Reynolds, R.M.; Rudolph, M.C.; MacLean, P.; Hernandez, T.L.; Friedman, J.E.; Krebs, N.F. Bioactive components in human milk are differentially associated with rates of lean and fat mass deposition in infants of mothers with normal vs. elevated BMI. *Pediatric obesity* 2018, *13*, 598-606, doi:10.1111/ijpo.12394.
65. Mohamad, M.; Loy, S.L.; Lim, P.Y.; Wang, Y.; Soo, K.L.; Mohamed, H.J.J. Maternal Serum and Breast Milk Adiponectin: The Association with Infant Adiposity Development. *International journal of environmental research and public health* 2018, *15*, doi:10.3390/ijerph15061250.
66. Grunewald, M.; Hellmuth, C.; Kirchberg, F.F.; Mearin, M.L.; Auricchio, R.; Castillejo, G.; Korponay-Szabo, I.R.; Polanco, I.; Roca, M.; Vriezinga, S.L.; et al. Variation and Interdependencies of Human Milk Macronutrients, Fatty Acids, Adiponectin, Insulin, and IGF-II in the European PreventCD Cohort. *Nutrients* 2019, *11*, doi:10.3390/nu11092034.
67. Aydin, S.; Aydin, S.; Ozkan, Y.; Kumru, S. Ghrelin is present in human colostrum, transitional and mature milk. *Peptides* 2006, *27*, 878-882, doi:10.1016/j.peptides.2005.08.006.
68. Kierson, J.A.; Dimatteo, D.M.; Locke, R.G.; Mackley, A.B.; Spear, M.L. Ghrelin and cholecystokinin in term and preterm human breast milk. *Acta paediatrica (Oslo, Norway : 1992)* 2006, *95*, 991-995, doi:10.1080/08035250600669769.
69. Aydin, S.; Geckil, H.; Karatas, F.; Donder, E.; Kumru, S.; Kavak, E.C.; Colak, R.; Ozkan, Y.; Sahin, I. Milk and blood ghrelin level in diabetics. *Nutrition* 2007, *23*, 807-811, doi:10.1016/j.nut.2007.08.015.
70. Ilcol, Y.O.; Hizli, B. Active and total ghrelin concentrations increase in breast milk during lactation. *Acta paediatrica (Oslo, Norway : 1992)* 2007, *96*, 1632-1639, doi:10.1111/j.1651-2227.2007.00493.x.
71. Aydin, S. The presence of the peptides apelin, ghrelin and nesfatin-1 in the human breast milk, and the lowering of their levels in patients with gestational diabetes mellitus. *Peptides* 2010, *31*, 2236-2240, doi:10.1016/j.peptides.2010.08.021.
72. Yis, U.; Ozturk, Y.; Sisman, A.R.; Uysal, S.; Soylu, O.B.; Buyukgebiz, B. The relation of serum ghrelin, leptin and insulin levels to the growth patterns and feeding characteristics in breast-fed versus formula-fed infants. *Turk J Pediatr* 2010, *52*, 35-41.
73. Karatas, Z.; Durmus Aydogdu, S.; Dinleyici, E.C.; Colak, O.; Dogruel, N. Breastmilk ghrelin, leptin, and fat levels changing foremilk to hindmilk: is that important for self-control of feeding? *European journal of pediatrics* 2011, *170*, 1273-1280, doi:10.1007/s00431-011-1438-1.
74. Savino, F.; Petrucci, E.; Lupica, M.M.; Nanni, G.E.; Oggero, R. Assay of ghrelin concentration in infant formulas and breast milk. *World journal of gastroenterology* 2011, *17*, 1971-1975, doi:10.3748/wjg.v17.i15.1971.
75. Savino, F.; Benetti, S.; Lupica, M.M.; Petrucci, E.; Palumeri, E.; Cordero di Montezemolo, L. Ghrelin and obestatin in infants, lactating mothers and breast milk. *Hormone research in paediatrics* 2012, *78*, 297-303, doi:10.1159/000345876.
76. Slupecka-Ziemilska, M.; Wolinski, J.; Herman, A.P.; Romanowicz, K.; Dziegielewska, Z.; Borszewska-Kornacka, M.K. Influence of preterm delivery on ghrelin and obestatin concentrations in maternal plasm, milk and their expression in mammary epithelial cells. *Journal of physiology and pharmacology : an official journal of the Polish Physiological Society* 2017, *68*, 693-698.

77. Young, B.E.; Patinkin, Z.; Palmer, C.; de la Houssaye, B.; Barbour, L.A.; Hernandez, T.; Friedman, J.E.; Krebs, N.F. Human milk insulin is related to maternal plasma insulin and BMI: but other components of human milk do not differ by BMI. *European journal of clinical nutrition* 2017, *71*, 1094-1100, doi:10.1038/ejcn.2017.75.
78. Shehadeh, N.; Khaesh-Goldberg, E.; Shamir, R.; Perlman, R.; Sujov, P.; Tamir, A.; Makhoul, I.R. Insulin in human milk: postpartum changes and effect of gestational age. *Archives of disease in childhood. Fetal and neonatal edition* 2003, *88*, F214-216, doi:10.1136/fn.88.3.f214.
79. Whitmore, T.J.; Trengove, N.J.; Graham, D.F.; Hartmann, P.E. Analysis of insulin in human breast milk in mothers with type 1 and type 2 diabetes mellitus. *Int J Endocrinol* 2012, *2012*, 296368, doi:10.1155/2012/296368.
80. Ellsworth, L.; Perng, W.; Harman, E.; Das, A.; Pennathur, S.; Gregg, B. Impact of maternal overweight and obesity on milk composition and infant growth. *Maternal & child nutrition* 2020, *16*, e12979, doi:10.1111/mcn.12979.
81. Ilcol, Y.O.; Hizli, Z.B.; Eroz, E. Resistin is present in human breast milk and it correlates with maternal hormonal status and serum level of C-reactive protein. *Clinical chemistry and laboratory medicine* 2008, *46*, 118-124, doi:10.1515/cclm.2008.019.
